# Hemodynamic effects of adjunct arginine vasopressin to norepinephrine in septic shock: insights from a prospective multicenter registry study

**DOI:** 10.1186/s13613-025-01472-w

**Published:** 2025-04-29

**Authors:** Max Melchers, Vivienne de Smet, Chrissie Rooijakkers, Jonathan Huising, Wander Vermeulen, Beyza Nur Nisa Köktaş, Karlijn Johanna van de Vusse, Kimia Milani Sabzewar, Shakti Bedanta Mishra, Carina Bethlehem, Dirk P. Boer, Nedim Cimic, Mirella van Duijnhoven, Tim Frenzel, Jordi Liesveld, Gianluca Paternoster, Susanne Stads, Jan J. Weenink, Barbara Festen-Spanjer, Peter Pickkers, Arthur Raymond Hubert van Zanten

**Affiliations:** 1 Department of Intensive Care, Gelderse Vallei Hospital, Ede, the Netherlands; 2 Division of Human Nutrition and Health, Wageningen University & Research, Wageningen, the Netherlands; 3 Department of Intensive Care, Siksha ‘O’ Anusandhan University Hospital, Bhubaneswar, Odisha India; 4 Department of Intensive Care, Medical Center Leeuwarden, Leeuwarden, the Netherlands; 5 Department of Clinical Pharmacy & Pharmacology, Medical Center Leeuwarden, Leeuwarden, the Netherlands; 6 Department of Intensive Care, Maasstad Hospital, Rotterdam, the Netherlands; 7 Department of Intensive Care, Tjongerschans Hospital, Heerenveen, the Netherlands; 8 Department of Intensive Care, Viecuri Medical Center, Venlo, the Netherlands; 9 Department of Intensive Care, Radboud University Medical Center, Nijmegen, the Netherlands; 10 Department of Intensive Care, Rivierenland Hospital, Tiel, the Netherlands; 11 Department of Health Science Anesthesia and ICU School of Medicine, University of Basilicata San Carlo Hospital, Potenza, Italy; 12 Department of Intensive Care, Ikazia Hospital, Rotterdam, the Netherlands; 13 Department of Intensive Care, Spaarne Gasthuis, Haarlem, the Netherlands; 14 Radboud Center for Infectious Diseases, Radboud University Medical Center, Nijmegen, the Netherlands

**Keywords:** Sepsis, Shock, Norepinephrine, Arginine vasopressin, Intensive care unit, Body mass index

## Abstract

**Background:**

The Surviving Sepsis Campaign guidelines suggest adding arginine vasopressin (AVP) when norepinephrine (NE) doses reach 0.25–0.50 µg/kg/min in septic shock patients. However, relying solely on a NE threshold has limitations, as other factors may be valuable in guiding AVP therapy during septic shock. Therefore, we aimed to identify additional patient characteristics associated with AVP hemodynamic responsiveness.

**Methods:**

A multicenter, prospective, observational study was conducted among adult ICU patients who met the predefined criteria for septic shock (not reaching the individual target mean arterial pressure despite adequate fluid resuscitation and NE base dose > 0.25 µg/kg/min) and received AVP therapy. AVP hemodynamic responsiveness was the primary study outcome, defined as stabilization or decrease of NE infusion rate two hours after initiating AVP. Secondary outcomes included shock duration and rebound hypotension following termination of AVP infusion. Univariate and multivariable regression analyses were performed to detect associations between characteristics and outcomes.

**Results:**

Between May 2020 and October 2023, 200 septic shock patients originating from 11 different ICUs were included. Of these, 153 (79%) met the definition for AVP hemodynamic responsiveness. Obesity and hyperlactatemia was negatively associated with AVP-response (adjusted Odds Ratio [aOR] 0.30, 95%CI 0.14–0.65 and aOR 0.86, 95%CI 0.75–0.99, respectively), while a NE infusion rate ≥ 0.30 µg/kg/min showed positive odds of AVP response (aOR 2.33, 95%CI 1.06–5.14). Incidence of new-onset atrial fibrillation was lower in AVP responders than non-responders (4% vs. 14%, *p* = 0.013). Higher body mass index (BMI) , NE infusion rate and duration prior to AVP initiation was associated with longer shock duration (aOR 1.06, 95%CI 1.02–1.11, aOR 1.12, 95%CI 1.01–1.25, and 1.01 95% CI 1.00–1.03, respectively), while higher pH associated with lower likelihood of prolonged shock (aOR 0.80, 95%CI 0.64–0.99). Rebound hypotension occurred in 9% when AVP was terminated, and AVP duration > 24 h was negatively associated with rebound hypotension (OR 0.22, 95%CI 0.05–0.85).

**Conclusions:**

Arterial lactate, pH, BMI, and NE duration and dose were associated with AVP responsiveness and shock duration during septic shock, and rebound hypotension occurred in 9% during recovery. Our findings suggest that beyond NE thresholds, specific factors could be considered to optimize adjunctive AVP therapy in septic shock patients.

**Graphical Abstract:**

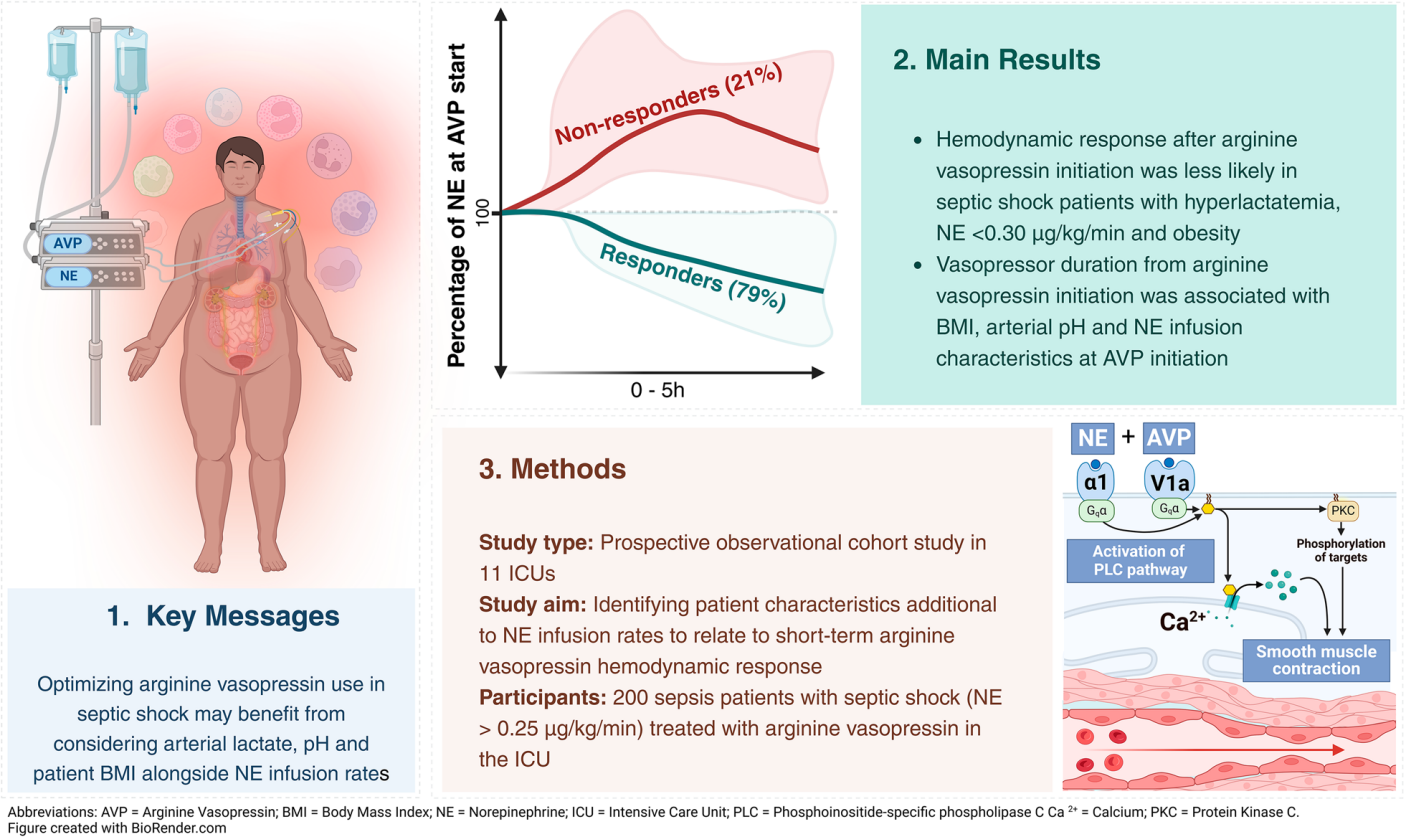

**Supplementary Information:**

The online version contains supplementary material available at 10.1186/s13613-025-01472-w.

## Background

Septic shock is the leading cause of death in Intensive Care Unit (ICU) patients. This medical emergency necessitates rapid fluid resuscitation and vasopressor therapy to restore vascular tone and maintain adequate organ perfusion. Despite advancements in sepsis management, mortality rates for septic shock remain high at 30–35% [1]. Norepinephrine (NE) is the first-line vasoactive agent for restoring mean arterial pressure (MAP) [2]. While it is acknowledged that an important proportion of patients may progress to catecholamine-resistant shock, this condition still lacks a clear-cut and uniform definition [3]. Higher infusion rates of NE are associated with mortality [4], possibly by inducing adverse effects, e.g. arrhythmias [5], or immunodysregulation [6]. Second-line agents for septic shock may reduce the catecholamine burden and mitigate these unfavourable effects.

Arginine vasopressin (AVP) is an endogenous peptide hormone that enhances vasoconstriction and water reabsorption in the kidneys [7]. The VANISH and VASST trials showed that AVP infusion reduces the dependency on catecholamines and it may therefore represent a valuable agent in the therapeutic armamentarium against septic shock [8, 9]. The Surviving Sepsis Campaign 2021 guidelines suggest starting AVP at a fixed infusion rate independent of weight (up to 0.03 IU/min) as an early adjunct when NE infusion rates reach 0.25–0.50 µg/kg/min [10, 11], a threshold for AVP initiation that still needs to be validated [12, 13]. In addition, these guidelines do not explicitly state whether this cut-off is expressed in NE base or salt formulation, an issue that is gaining in attention [14]. Nevertheless, focusing exclusively on a NE threshold for AVP initiation may have shortcomings, as it appears plausible that other patient-dependent factors may also play a role [15]. Currently, factors that account for the variability in patient hemodynamic responsiveness to AVP are largely unclear. While NE dose increments could help identify vasodilatory shock patients who may benefit from multimodal vasopressor therapy regarding decatecholaminization and shock duration [11], its utility in guiding decision-making remains unclear.

Septic shock is associated with endogenous vasopressin deficiency; however, plasma vasopressin levels appear not to be associated with hemodynamic responsiveness to AVP [16]. Several studies have focused on patient characteristics related to AVP responsiveness 4–6 h post-initiation [13, 17, 18], while more rapid efficacy is expected based on AVP pharmacodynamics [7], and beneficial effects may be expected within 4 h of initiation [12]. In addition, combining MAP and NE requirements to define responsiveness may be inappropriate in observational data since the desired MAP may vary in the acute phase based on organ perfusion signs. Moreover, decatecholaminization is currently considered among the primary goals of adjunctive vasopressor agents in septic shock [19].

Early initiation of AVP in septic shock has been proposed to reduce shock duration [12, 20]. However, it still remains uncertain to what extent NE infusion rate and duration influences vasopressor dependency. Additionally, tapering NE before reducing the AVP infusion rate could potentially prevent rebound hypotension [21, 22], although this approach is not consistently implemented, and the influence of other factors that mitigate the risk of rebound hypotension during recovery remains unclear.

Therefore, the primary aim of the current study was to identify patient characteristics additional to NE infusion rates to relate to short-term AVP hemodynamic responsiveness in patients with septic shock.

## Methods

### Study population and design

A multicenter, prospective, observational study was conducted among adult (≥ 18 years) critically ill patients that met the predefined Sepsis-3 criteria (known or suspected infection with new organ dysfunction defined by a change of sequential organ function assessment (SOFA) of at least 2 points) and shock (persistent hypotension/not reaching the individual target MAP despite adequate fluid resuscitation (> 30 mg/kg IV fluid in 3 h [10]) and NE base equivalent dose > 0.25 µg/kg/min, which aligned with institutional practices to initiate AVP across participating centers), and received AVP as adjunct vasopressor. Patients were excluded in case of acute myocardial, mesenterial or digital ischemia before the initiation of AVP. Other exclusion criteria were chronic renal replacement therapy (RRT), treatment limitation other than do-not-resuscitate, medical history of vasculitis, moribund (defined as an expected survival duration < 48 h), admission for burn wounds, and second ICU admission during the same hospital admission. Furthermore, patients with insufficient NE data to determine the study's primary outcome (i.e. no infusion rate recorded at the time of AVP initiation or during follow-up, or recorded only once daily) were excluded from the analysis. Due to the study's observational nature, the study received a waiver from the Dutch ‘Medical Research Involving Human Subjects Act' from the medical ethical committee of Wageningen University and Research on December 20, 2020.

#### AVP guideline

All participating centers received a guideline from the coordinating center (Supplemental Fig. 1) in which was described that AVP should be initiated at 0.01 IU/min when the NE infusion rate exceeded 0.25 µg/kg/min. If the target MAP was not achieved within 15–20 min, the AVP dose was increased with steps of 0.01 IU/min up to a maximum infusion rate of 0.03 IU/min. AVP was titrated independently of body weight. When hemodynamics improved (i.e. NE ≤ 0.10 µg/kg/min), AVP was tapered before NE was stopped with steps of 0.01 IU/min every 20 min until stop. This approach aligns with previous findings [21], may reduce costs and provided the ability to treat non-septic shock related hypotension (i.e. due to mechanical ventilation with positive end-expiratory pressure and/or sedation) during the recovery phase with NE. However, due to the study's observational nature, participating centers and treating physicians could deviate from this guideline. This implies deviation NE threshold to initiate and taper AVP, as well as from the starting dose and titration speed of AVP.

**Fig. 1:**
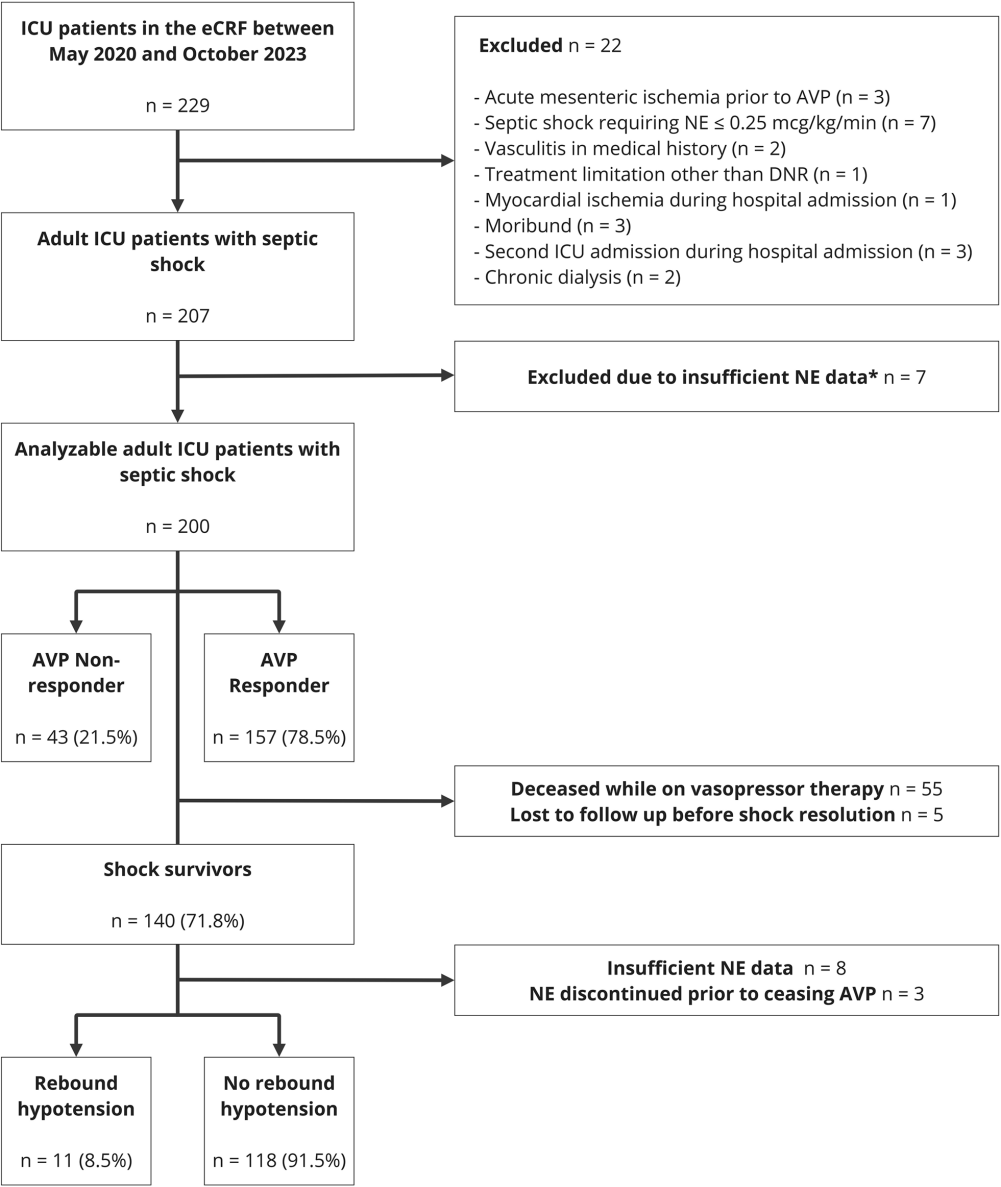
Flowchart of the study population. * No NE infusion rate registered at AVP initiation (n = 4), NE infusion rate only registered once daily (n = 2) or NE infusion rate was only registered at AVP initiation but not during follow-up (n = 1). *ICU* Intensive Care Unit, *eCRF* electronic case report form, *DNR* Do-Not-Resuscitate, *AVP* Arginine vasopressin, *NE* Norepinephrine

#### Data collection

Data were extracted by study sites from electronic medical records and entered in Castor’s EDC ® (Ciwit B.V., Amsterdam, The Netherlands) following the acquisition of informed consent from the patient or, when applicable, from a legal representative, next of kin, or proxy. Patient characteristics include demographics, comorbidities, shock-related characteristics (i.e. SOFA score, acute physiological and chronic health evaluation (APACHE IV) score, organ support), and sepsis-related characteristics (i.e. infection site and culture results). NE duration, vital signs, net fluid balance (fluid input minus fluid output recorded from ICU admission) and laboratory values were recorded upon baseline, defined as the start of AVP infusion. Vital signs and laboratory values were also recorded at 1, 3, 5, 12 and 24 h after AVP initiation. Laboratory values within 1 h and closest to these timepoints were used for data analysis. NE and AVP infusion rate changes were recorded from start to end of vasopressor therapy. NE equivalent (NEE) dosages were calculated as described previously, with AVP dosage multiplied by 2.5 to obtain the NEE [2]. Delta NE and NEE on study time points were expressed as percentage from baseline NE infusion rate. Clinical data registration was stopped if the patient left the ICU, died, or AVP was stopped for 24 h. A stepwise reduction of AVP in one hour to stop was considered as tapering. Only the first period was registered if patients received a second treatment course with AVP during ICU admission. Data collection was monitored remotely and locally by the coordinating center's research staff.

#### Outcomes

The primary outcome of the study was AVP hemodynamic responsiveness. Currently, there is no universally accepted definition for “hemodynamic response”. The investigators prospectively determined this criterion by taking into account the exponential NE requirement during catecholamine-resistant septic shock [11] and the short-acting pharmacologic efficacy of AVP [7]. Therefore, AVP hemodynamic responsiveness was defined as a stabilization or decrease of NE-infusion rates two hours after initiating AVP independent of its starting dose. Secondary outcomes included shock duration, which was defined as the vasopressor-dependent time from the start of AVP, and rebound hypotension, defined as an increase in adjuvant NE dose [24] within two hours after stopping AVP. In addition, other clinical outcomes and adverse events, including arrythmia’s, new-onset mesenteric, digital, and myocardial ischemia, and hyponatremia (sodium < 130 mmol/L), were documented and compared between AVP responders and non-responders. The local investigator evaluated the likelihood of a relationship between AVP administration and the occurrence of these adverse events. Survival was monitored for up to 180 days following ICU admission.

### Statistical analyses

#### Demographics and characteristics

Relevant patient demographics, clinical characteristics and additional outcome parameters were extracted from the eCRF and compared between responders and non-responders. Continuous variables were displayed as means with standard deviation (SD) if normally distributed and as medians with interquartile range [IQR] otherwise. Normality of distribution was tested by inspecting histograms and additional Kolmogorov–Smirnov-tests. Student's t-test or Mann–Whitney U-test assessed differences for continuous variables. Missingness completely at random (MCAR) was tested using Hawkin’s test for non-parametric baseline continuous variables with missing data. MCAR data with less than 40% missingness was imputed using multiple imputation by chained equations using predictive mean matching, generating five datasets. Categorical variables were presented as numbers with percentages, and their differences were tested by the Chi-squared test or Fisher's exact test in case cells expected count was less than 5. Body mass index (BMI) was grouped into obesity (BMI > 30 kg/m^2^) and non-obesity (BMI ≤ 30 kg/m^2^). In addition, baseline NE infusion was split into < 0.30 and ≥ 0.30 µg/kg/min since a subset of participating centers utilised NE 0.30 µg/kg/min as threshold for AVP initiation instead of NE 0.25 µg/kg/min.

#### Outcomes

Univariate and multivariable binary logistic regression analyses were performed with the imputed dataset to detect associations between characteristics and the presence of AVP hemodynamic responsiveness in the whole cohort and rebound hypotension in shock survivors. A sensitivity analysis was conducted for characteristics associated with a decrease in NE infusion rate two hours after AVP initiation. Youden’s receiver operating characteristic (ROC) curve indices were obtained to identify optimal NE dose cut-off values to predict AVP responsiveness within the cohort. Wilcoxon signed-rank test was used to compare repeated measures within groups. Univariate linear mixed models were conducted to detect between-group differences in dynamic parameters MAP, arterial pH and lactate, net fluid balance following AVP initiation, and NE requirement pre-AVP by adding the interaction between response and time unit as a fixed effect. In case of significant heteroscedasticity, robust standard errors were presented. Sensitivity checks were performed in case of significant influential points. Proportional hazard regression analyses were used to obtain associations between characteristics and the probability of prolonged shock duration in shock survivors. To identify covariates for inclusion in the multivariable models, Least Absolute Shrinkage and Selection Operator (LASSO) regression was applied with covariates selected at the optimal penalty parameter that minimized cross validation (λ_min_). Covariates were excluded in case of multicollinearity (e.g. variance of inflation factor > 5.0) or when the proportional hazard assumption was violated (e.g. inspection of Shoenfeld Residuals Plot). Statistical analyses were conducted using SPSS Statistics software (IBM Corp. Version 29.0 Armonk, NY, USA) and R studio (Version 2023.03.0, Posit Software, PBC, 2022). Graphs were created using R studio (Version 2023.03.0, Posit Software, PBC, 2022).

## Results

### Study population

Between May 2020 and October 2023, 229 subjects were registered in the eCRF. Of these, 22 were excluded due to exclusion criteria and seven due to insufficient NE data. The 200 included adult patients with septic shock were treated in 11 different centers, of which one was in India, one in Italy, and the remaining in the Netherlands. During data analysis it appeared that one center reported in NE tartrate equivalent dose resulting in NE base equivalent dose < 0.25 mcg/kg/min in three subjects that were included in the analysis. In seven (64%) centers, a NE threshold of 0.25 µg/kg/min was used to initiate AVP; in two (18%) centers, a NE threshold of 0.30 µg/kg/min, and in the remaining two (18%) this was variable. The difference between the predefined NE threshold and dosage upon which AVP was initiated was 0.16 [0.05–0.30] µg/kg/min higher than their protocolized NE threshold for AVP (n = 188 patients).

### AVP hemodynamic responsiveness

One hundred fifty-seven (79%) septic shock patients met the predefined criteria for AVP hemodynamic responsiveness (Fig. 1; an expanded flowchart is presented in Supplemental Fig. 2). Baseline characteristics of AVP responders and non-responders are depicted in Table 1 and missing baseline data are presented in Supplemental Fig. 3. At baseline, AVP responders had lower arterial lactate levels and a lower BMI compared to non-responders. Furthermore, higher baseline NE infusion rates were observed in responders compared to non-responders. NE dosage cut-off to predict AVP response in this cohort was ≥ 0.34 µg/kg/min (AUC 0.64, 95%CI 0.54–0.73). No baseline differences between responders and non-responders were observed regarding SOFA-score, cardiac index or resuscitation status. In addition, there was no difference between responders and non-responders in delta NEE throughout the five hours prior to AVP initiation (*p* = 0.716).

**Table 1:** Baseline characteristics of responders and non-responders

Characteristics	Total *n* = 200	Non-responder^e^ *n* = 43	Responder^e^ *n* = 157	*p*-value
Age, years	67 [57–75]	66 [55–73]	67 [58–75]	0.344
Sex, male	112 (56.0)	24 (55.8)	88 (56.1)	0.978
BMI on admission, kg/m^2^	27.0 [24.0–31.8]	30.4 [26.8–34.8]	26.1 [23.7–30.9]	< 0.001
Obesity^a^, n (%)	64 (32.2)	23 (53.5)	41 (26.3)	< 0.001
IBW^b^, kg	58.3 [47.8–66.7]	59.2 [50.8–71]	57.7 [36.9–66.4]	0.117
Comorbidities, n (%)				
COPD	17 (8.5)	1 (2.3)	16 (10.2)	0.129
Diabetes	37 (18.5)	10 (23.3)	27 (17.2)	0.365
Heart failure	13 (6.5)	2 (4.7)	11 (7.0)	0.739
Kidney disease	12 (6.0)	3 (7.0)	9 (5.7)	0.723
Malignant disease	17 (8.5)	5 (11.6)	12 (7.6)	0.406
Immunocompromised^c^	18 (9.0)	4 (9.3)	14 (8.9)	0.938
Recent anti-hypertensive use^d^, n (%)				
ACE-inhibitor	35 (21.1)	17 (17.9)	30 (22.1)	0.579
Calcium-channel inhibitor	21 (12.8)	3 (7.7)	19 (14.3)	0.278
ARB	12 (7.0)	1 (2.6)	11 (8.3)	0.219
Beta-blocker	40 (23.8)	9 (23.1)	32 (24.1)	0.899
Infection site, n (%)				
Pulmonary	62 (31.0)	10 (23.3)	52 (33.1)	0.215
Abdominal	94 (47.0)	24 (55.8)	70 (44.6)	0.191
Urinary tract	11 (5.5)	2 (4.7)	9 (5.7)	0.783
Central nervous system	2 (1.0)	0 (0)	2 (1.3)	0.457
Skin and soft tissue	22 (11.0)	5 (11.6)	17 (10.8)	0.882
Other	9 (4.5)	2 (4.7)	7 (4.5)	0.957
Pathogen, n (%)				
Gram stain positive	55 (27.6)	14 (32.6)	41 (26.3)	0.415
Gram stain negative	52 (26.1)	9 (20.9)	43 (27.6)	0.381
Gram stain mixed	23 (11.6)	5 (11.5)	18 (11.6)	0.987
Viral/parasites	12 (6.0)	1 (2.3)	11 (7.1)	0.468
Culture negative	57 (28.6)	14 (32.6)	43 (27.6)	0.521
Shock related characteristics				
APACHE IV score	92 [75–112]	93 [77–117]	92 [75–111]	0.555
SOFA score	10 [8–12]	10 [8–12]	10 [8–12]	0.926
Mechanical Ventilation, n (%)	165 (82.5)	34 (79.1)	131 (83.4)	0.504
RRT, n (%)	17 (8.5)	1 (2.3)	16 (10.2)	0.129
Creatinine, µmol/L	102 [74–166]	100 [72–178]	105 [76–166]	0.921
Net fluid balance, mL/kg	59 [34–118]	63 [35–106]	58 [34–128]	0.790
Received corticosteroids, n (%)	120 (60.0)	26 (60.5)	94 (59.9)	0.944
Received hydrocortisone, n (%)	101 (50.5)	23 (53.5)	78 (49.7)	0.498
Received intravenous calcium, n (%)	18 (12.4)	3 (9.1)	15 (13.4)	0.510
ScvO^2^, %	72 [64–78]	74 [67–81]	72 [64–78]	0.451
Cardiac Index, L/min/m^2^	2.7 [2.4–3.8]	2.7 [2.3–3.7]	2.7 [2.3–3.9]	0.986
Arterial lactate, mmol/L	3.2 [2.1–5.3]	4.2 [2.6–7.2]	2.9 [2.1–4.5]	0.027
Arterial pH	7.29 [7.22–7.34]	7.30 [7.24–7.34]	7.29 [7.22–7.33]	0.544
NE duration, hours	7 [3–17]	6 [3–12]	8 [4–18]	0.231
NEE delta 2 h prior to baseline, %	17 [6–39]	19 [6–46]	16 [6–39]	0.732
NE dose, µg/min	35.0 [26.7–46.7]	31.7 [25.0–46.7]	35.0 [28.3–46.7]	0.219
NE dose, µg/kg/min	0.42 [0.31–0.56]	0.33 [0.28–0.50]	0.44 [0.33–0.57]	0.005
NE ≥ 0.30 µg/kg/min, n (%)	155 (77.5)	26 (60.5)	129 (82.2)	0.003
AVP starting dose, IU/min	0.01 [0.01–0.02]	0.01 [0.01–0.01]	0.01 [0.01–0.02]	0.745

*BMI* Body Mass Index, *IBW* Ideal Body Weight, *COPD* Chronic Obstructive Pulmonary Disease, *ACE* Angiotensin Converting Enzyme, *ARB* Angiotensin Receptor Blocker, *APACHE* Acute Physiology and Chronic Health Evaluation, *SOFA* Sequential Organ Failure Assessment, *RRT* Renal Replacement Therapy; *ScvO*_*2*_ Central Venous Oxygen Saturation; *NE* Norepinephrine; *NEE* Norepinephrine Equivalent, *AVP* Arginine vasopressin; Missing baseline data are displayed in Supplemental Fig. 2

^a^BMI ≥ 30 kg/m^2^

^b^Obtained using Gallagher’s formula (25)

^c^Long term use of immunosuppressive therapy or use of corticosteroids (e.g. > 5 days 1 mg/kg prednisone or 20 days ≥ 0.1 mg/kg) or active chemo-or radiation therapy last year, or treatment for a lymphoma any time before ICU admission or documented humoral or cellular deficiencies

^d^ ≤ 48 h of ICU-admission

^e^Response was defined as a stabilization or decrease in NE requirement 2 h after AVP initiation

**Fig. 2:**
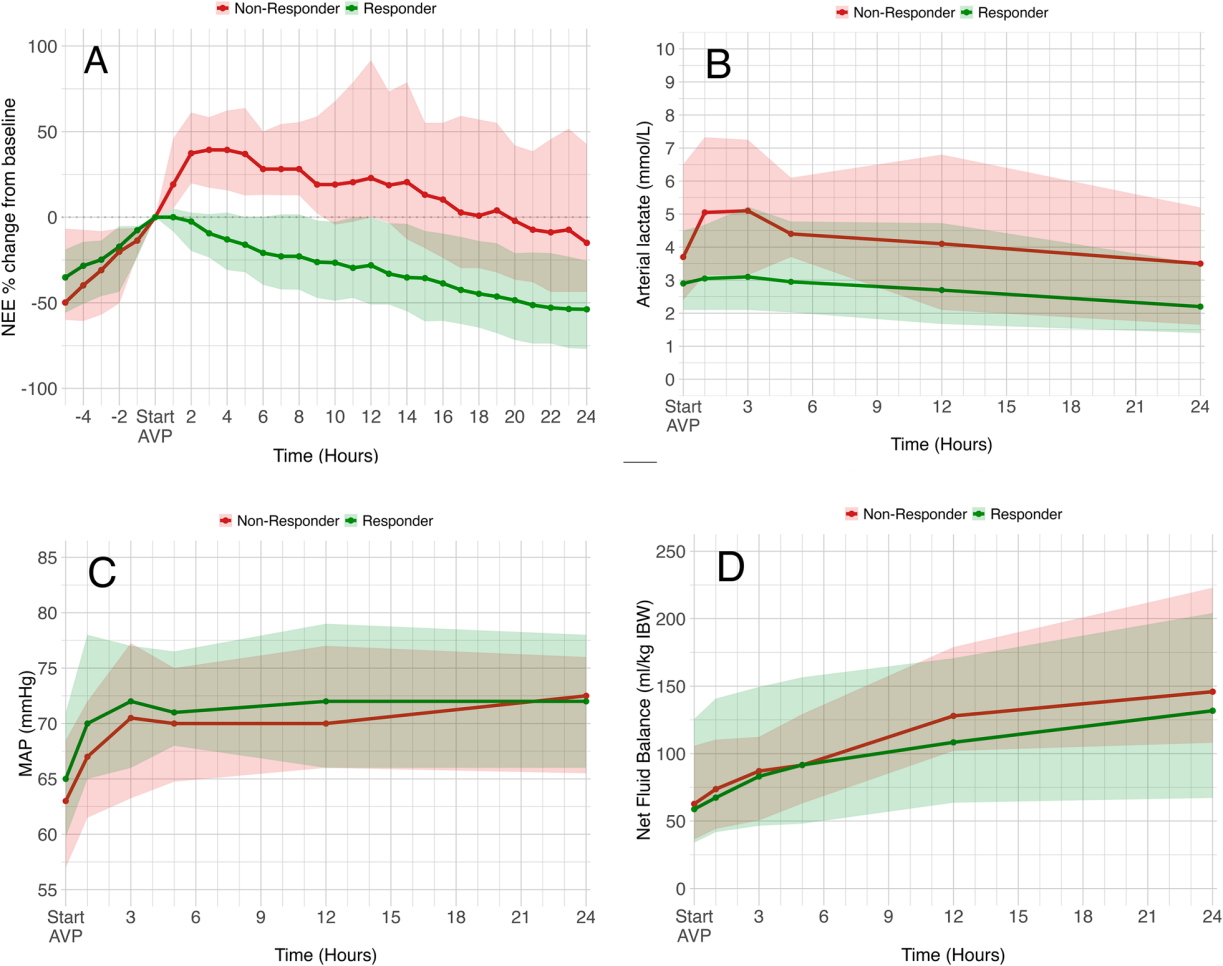
**Comparison of dynamics between AVP responders and non-responders.** **A**. **NEE dynamics before and after start of AVP.** A LMM was conducted applying robust standard errors to evaluate the difference in NEE dynamics between responders and non-responders during the five hours prior to AVP initiation. The change in NEE was not significantly different between responders and non-responders (*p* = 0.716). **B**. **Arterial lactate levels at baseline and after start of AVP.** A LMM showed responders had lower baseline lactate (*p* = 0.018), but lactate levels did not change significantly over time (*p* = 0.517), nor indicated different trajectories between responders and non-responders over time (*p* = 0.980). **C**. **Mean Arterial Pressure (MAP) at baseline and after start of AVP. **Results of LMM showed a significantly higher baseline MAP in responders compared to non-responders (*p* = 0.005) and significant increase in MAP over time overall (*p* < 0.001), but similar trajectory among responders and non-responders (*p* = 0.075). After applying robust standard errors due to heteroscedasticity the baseline difference remained significant (*p* = 0.029) with an overall significant increase over time (*p* = 0.015), but no significant interaction effect with response (*p* = 0.170), indicating similar MAP trajectories between groups. **D**. **Net fluid balance at baseline and after start of AVP.** A LMM showed no significant difference at baseline net fluid balance between responders and non-responders (*p* = 0.993). Non-responders accumulated fluid at an average rate of 1.5 ml/kg IBW per hour more than responders (*p* < 0.001), which remained significant after applying robust standard errors (*p* = 0.021) due to heteroscedasticity. In all figures medians alongside interquartile ranges are presented. *NEE* Norepinephrine Equivalent, *AVP* Arginine vasopressin, *MAP* Mean Arterial Pressure, *IBW* Ideal Body Weight, *LMM* Linear Mixed Model

Imputed continuous baseline data is presented in Supplemental Table 1. In multivariable logistic regression analysis, obesity and hyperlactatemia were negatively associated with AVP response (adjusted Odds Ratio [aOR] 0.30, 95%CI 0.14–0.65 and aOR 0.86, 95%CI 0.75–0.99, respectively), while a NE infusion rate ≥ 0.30 µg/kg/min was associated with AVP response (aOR 2.33, 95%CI 1.06–5.14; Supplemental Table 2).

**Table 2:** Clinical outcomes of study patients

Outcome parameters	Total, *n* = 200	Non-responder *n* = 43	Responder *n* = 157	*p*-value
New-onset atrial fibrillation^a^, n (%)	12 (6)	6 (14)	6 (4)	0.013
Mesenteric ischemia, n (%)	5 (3)	0	5 (3)	0.586
Acute myocardial ischemia, n (%)	4 (2)	1 (2)	3 (2)	1.000
Digital ischemia, n (%)	5 (3)	1 (2)	4 (3)	1.000
New-onset hyponatremia^b^, n (%)	2 (1)	1 (2)	1 (1)	0.385
Total AVP duration^c^, hours	30 [17–49]	29 [20–48]	30 [16–51]	0.877
Cumulative AVP dose^d^, IU	34.8 [20.1–69.4]	37.9 [23.9–74.3]	34.0 [19.8–68.6]	0.548
Rebound hypotension^e^, n (%)	13 (9)	3 (12)	10 (9)	0.704
Survived shock^f^, n (%)	140 (72)	28 (68)	112 (73)	0.575
Shock duration, hours	64 [36–101]	65 [36–103]	64 [36–100]	0.807
MV duration^g^, hours	126 [34–252]	178 [28–266]	119 [37–246]	0.734
MV duration in survivors^h^, hours	174 [73–346]	233 [132–401]	134 [68–344]	0.080
RRT duration^i^, hours	124 [55–233]	58 [22–94]	136 [63–287]	0.037
RRT duration in survivors^j^, hours	153 [74–287]	71 [54–132]	170 [103–329]	0.069
ICU mortality, n (%)	71 (38)	16 (40)	55 (37)	0.720
ICU LOS^k^, days	8 [3–17]	10 [3–20]	7 [3–16]	0.281
ICU LOS in survivors^l^, days	10 [6–20]	14 [9–26]	10 [6–19]	0.049
Hospital LOS^m^, days	18 [7–32]	17 [6–36]	19 [7–31]	0.918
90 days mortality, n (%)	87 (44)	20 (50)	67 (43)	0.677
180 days mortality, n (%)	89 (45)	20 (50)	69 (44)	0.790

*AVP* Arginine vasopressin, *MV* Mechanical Ventilation, *RRT* Renal Replacement Therapy, *ICU* Intensive Care Unit, *LOS* Length of Stay; *R* Responder; *NR* Non-responder

^a^Within 12 h of AVP initiation

^b^Sodium ≤ 130 mmol/L

^c^Missing in 2 (0 NR; 2 R)

^d^Missing in 8 (5 NR; 3 R)

^e^ Increase in NE infusion rate 2 h after ceasing AVP therapy. Missing in 56 (NR 17; 39 R), of which in 40 due to mortality while receiving AVP and 4 due to NE stopped before AVP

^f^Missing in 5 (2 NR; 3 R) due to transferral to another ICU before shock resolution

^g^177 patients received mechanical ventilation

^h^115 patients (27 NR; 88 R) after excluding patients who died on mechanical ventilation

^i^36 patients (6 NR; 30 R) received RRT after AVP initiation

^j^26 patients (4 NR; 22 R) after excluding patients who died while on RRT (n = 7) or information was missing (n = 1)

^k^Missing in 3 (0 NR; 3 R)

^l^in 115 ICU survivors (24 NR; 94 R)

^m^Missing in 5 (1 NR; 4 R)

Among AVP responders, 91 (57%) exhibited a decrease in NE requirements and in 66 (43%) there was a stabilization of NE requirements 2 h after AVP initiation. In the sensitivity analysis, a NE infusion rate ≥ 0.30 µg/kg/min alongside higher age were associated with a reduction in NE requirement 2 h after AVP initiation (aOR 5.12, 95%CI 2.10–12.53 and aOR 1.03, 95% CI 1.01–1.06, respectively), while obesity and hyperlactatemia were no longer significantly associated (aOR 0.57, 95% CI 0.29–1.14 and aOR 0.89, 95%CI 0.78–1.01, respectively; Supplemental Table 3).

While responders had higher NE infusion rates at baseline compared to non-responders, NE and NEE dose were significantly lower in responders compared to non-responders two hours after AVP initiation (0.36 [0.28–0.50] vs. 0.49 [0.36 vs. 0.68] µg/kg/min, and 0.41 [0.33–0.55] vs. 0.54 [0.43–0.73] µg/kg/min, respectively, both *p* < 0.001) (Supplemental Figs. 4 & 5).

### Follow-up on AVP responders

The dynamics of NEE infusion rate from baseline (%), arterial lactate, MAP and net fluid balance of AVP responders and non-responders in the 24 h following AVP initiation, including NEE 5 h prior to AVP initiation, are depicted in Table 2. Dynamics of these repeated measures in patients who achieved NE reduction compared to those with NE stabilization or non-response at two hours after start of AVP is displayed in Supplemental Fig. 6. The dynamics in NE and NEE in responders, non-responders and within the whole cohort as percentage of baseline are presented in Supplemental Figs. 7 & 8. Arterial pH decreased more per hour in non-responders compared to responders in the five hours following AVP initiation (average 0.01, *p* = 0.05; Supplemental Fig. 9). Fig. 2 displays clinical outcomes of AVP responders and non-responders. Non-responders developed atrial fibrillation within twelve hours after initiation of AVP treatment more frequently than responders (14% vs. 4%, *p* = 0.013), which was associated with ICU mortality (OR 3.62, 95%CI 1.05–12.49) in logistic regression. RRT duration was significantly longer in responders compared to non-responders (134 [68–344] vs. 58 [22–94] hours, *p* = 0.037). After exclusion of patients who died on RRT this difference lost statistical significance.

### Shock duration

Of the included patients, 5 patients were lost to follow-up for shock duration analysis due to transferal to another ICU before shock resolution. Eventually, 55 (28%) patients died before shock resolution, and 140 (72%) survived septic shock (Fig. 1). The latter had a median [IQR] AVP duration of 31 [20–48] hours and a median [IQR] vasopressor duration of 66 [44–102] hours. In univariate analysis in shock survivors, baseline NE duration (OR 1.02, 95%CI 1.00–1.03) and infusion rate (µg/min, OR 1.02 95%CI 1.01–1.03), BMI (OR 1.03 95%CI 1.01–1.06), arterial pH (per 0.1 increase OR 0.73, 95%CI 0.59–0.89), and net fluid balance (per liter OR 1.06, 95%CI 1.02–1.11) were significantly associated with prolonged shock duration after AVP initiation, while AVP responsiveness was not (OR 0.86, 95%CI 0.57–1.31). In the main proportional hazard regression model, NE duration and infusion rate at AVP initiation, and BMI significantly increased the probability of a prolonged vasopressor requirement from AVP initiation (per hour aOR 1.01, 95%CI 1.00–1.03, per 0.1 mcg/kg/min increase aOR 1.12, 95%CI 1.01–1.25, and per kg/m^2^ aOR 1.06, 95%CI 1.02–1.11, respectively), while higher baseline arterial pH was associated with a negative odd’s of prolonged shock duration (aOR 0.80, 95%CI 0.64–0.99; Supplemental Table 4).

### Rebound hypotension

AVP was discontinued in 133 (95%) because the patient had stabilized, in one (0.8%) due to lack of expected effect, in one (0.8%) due to an adverse event (digital ischemia), and in five (4%) due to an arbitrary maximum protocolized AVP duration (48 h). In 11 shock survivors, the occurrence of rebound hypotension could not be evaluated, as in 3 patients NE was discontinued prior to stopping AVP and in 8 insufficient NE data were available (Fig. 1). In the remaining 129 shock survivors, rebound hypotension occurred in 11 (9%) patients after discontinuation of AVP, with a median [IQR] NE increase of 33 [25–107] % two hours after AVP discontinuation. AVP duration more than 24 h was negatively associated with rebound hypotension (OR 0.22 95%CI 0.05–0.85). In addition, age was associated with rebound hypotension (OR 1.07 95%CI 1.00–1.14). Tapering of AVP was done in 134 (96%) of shock survivors but was not associated with rebound hypotension (0.45, 95%CI 0.05–4.20, *p* = 0.481). Both NE infusion rate when stopping AVP and being an AVP-responder were not associated with the occurrence of rebound hypotension (per 0.1 OR 0.92, 95%CI 0.59–1.42, and OR 0.99, 95%CI 0.98–1.01, respectively; Supplemental Table 5).

### Adverse events

The following adverse events judged to be possibly or probably related to the use of AVP occurred: digital ischemia in five (2.5%) patients, of which four possibly and one probably related to AVP; myocardial ischemia in one (0.5%) patient possibly related to AVP, hyponatremia in two (1%) patients possibly related to AVP, and mesenteric ischemia in two (1%) patients possibly related to AVP. One (0.5%) patient developed compartment syndrome of the underarm contralateral to the infusion site, judged as possibly related to AVP infusion. No adverse events definitely related to AVP usage occurred in this cohort.

## Discussion

Among 200 patients with septic shock and treated with AVP as a second-line vasopressor in 11 different ICUs, we found a hemodynamic response rate two hours after AVP initiation of 79% with an overall significant decrease in NE and NEE requirements. Obesity, NE infusion rate < 0.30 mcg/kg/min, and hyperlactatemia were negatively associated with AVP responsiveness, and high NE infusion rate and longer duration at AVP initiation alongside higher BMI and lower baseline arterial pH were associated with prolonged shock duration in shock survivors. Rebound hypotension occurred in 9% after ceasing AVP before NE.

### AVP response

The higher response rates to AVP in this study compared to previous reports may be attributed to differences in definitions. Earlier studies reported response-rates of 45% and 51% at six hours post-AVP initiation, defining response as maintaining MAP ≥ 65 mmHg with reduced catecholamine dosages [13, 18]. Jakowenko et al. reported 24% responders using a stricter definition of ≥ 50% NE reduction while maintaining MAP at four hours post-AVP initiation [17]. Conversely, another cohort study reported an AVP response rate of 81% in septic shock patients using similar criteria [27]. In clinical practice, defining hemodynamic response based solely on vasopressor requirements may be more appropriate, given the variability in target MAP during septic shock treatment [28, 29]. Furthermore, in severe septic shock, the rapidly increasing NE requirement should also be considered when defining AVP response [11]. Marking stabilization of NE requirement as AVP response in progressive septic shock seems appropriate and has led to a high response rate since a decrease in NE requirement occurred in 46%. Additionally, the short-acting pharmacological effects of AVP likely contributed to the higher response rates when evaluation is performed at two hours after initiation, whereas at later stages NE dose may also have increased as a result of the course of septic shock. Ragoonanan et al. agreed that the beneficial effects of AVP on shock may peak within the first four hours of initiation and then taper off [12].

Although AVP responsiveness at two hours appeared independent of AVP starting dose, we did observe an association between baseline NE infusion rate and the probability of AVP responsiveness which parallels the results by previous studies [12, 13]. Its association with a decrease in NE requirement was even more pronounced and it may indicate the development of catecholamine-resistance, highlighting the need for an adjunct vasopressor [3]. However, in the context of our study, where patients were only eligible for inclusion with an NE dosage > 0.25 µg/kg/min, it is crucial to note the potential consequences of delays in AVP initiation. We observed a median NE dosage 0.16 µg/kg/min above the protocolized thresholds at the time of AVP initiation. Given the higher likelihood of AVP responsiveness at elevated baseline NE infusion rates, these delays may paradoxically enhance the response to AVP by initiating it when catecholamine resistance is more pronounced, potentially increasing the clinical relevance of AVP in such cases. However, contrasting NE infusion rate at baseline, we did not observe significant between-group differences in NEE dynamics pre-AVP initiation as was previously proposed [11].

Similar to previous work, low baseline arterial lactate increased the probability of AVP responsiveness in our study [13, 30], and non-responders exhibited persistently elevated lactate levels over the first 24 h. However, in the sensitivity analysis, the relation between lactate and reduction in NE was no longer significant. A re-analysis of the VASST trial found improved survival rates with early AVP initiation at lactate levels ≤ 2 mmol/L [31]. Additionally, a difference in dynamics of arterial pH following AVP initiation was observed, suggesting variations in metabolic profiles between AVP responders and non-responders. Although we did not find between-group differences in arterial pH at baseline aligning with previous papers [17, 30], higher arterial pH increased the probability of more rapid shock resolution. Intra- and extracellular acidosis reduce vasopressin-induced vascular smooth muscle cell contraction by decreasing its affinity for the V1a receptor [30, 32]. Combined with NE requirements, low arterial lactate and high pH appear valuable markers for initiating AVP, reflecting less severe septic shock.

An association between BMI and hemodynamic response to AVP alongside prolonged shock duration was identified in this cohort which is in line with a previous observation [33]. The vasopressin system plays a key role in regulating metabolic pathways, including carbohydrate and lipid metabolism [34]. V1aR knockout models, which lack the receptor on which AVP exerts its vasoconstrictive abilities, are prone to obesity [35]. A deficiency or dysfunction of V1aR in obese patients could potentially contribute to their reduced hemodynamic response to AVP. Second, in septic shock, obese patients may receive suboptimal exposure to AVP with conventional dosing strategies due to its rapid distribution into extracellular fluid, and volume of distribution being affected by weight-based fluid resuscitation and increased adipose tissue [36, 37]. A post-hoc analysis of the VASST trial detected lower serum vasopressin levels after AVP initiation in obese patients compared to BMI < 25 kg/m^2^ [38]. Still, in retrospective analyses, no correlations were found between BMI-adjusted AVP dosing and catecholamine reduction or shock duration [36], nor was there a benefit of a higher fixed AVP starting dose in obese patients [36, 37]. In addition, obesity was no longer associated with a decrease in NE requirement in our sensitivity analysis. Prospective studies are required to elucidate whether BMI or body composition should be considered when initiating and dosing AVP in septic shock patients.

The synergistic efficacy of AVP in combination with corticosteroids remains a topic of debate given the complex physiologic interactions between these agents, and existing studies have reported conflicting results [8, 17, 27]. In our analysis, administering corticosteroids prior to AVP therapy in septic shock patients showed neither a significant improvement in responsiveness nor an impact on shock duration, indicating no discernible short-term benefit or harm from this combination.

Although ICU LOS in ICU survivors and the incidence of new-onset atrial fibrillation–explained by a higher catecholamine burden in non-responders and associated with poor outcomes in septic shock [5, 39]–were significantly lower in responders, there was no significant difference in ICU survival between the groups. This contrasts previous reports [13, 17], suggesting our sample size or hemodynamic response definition may have been insufficient to identify differences in survival rates. Still, two randomized controlled trials also failed to detect a significant benefit of AVP on survival in septic shock patients [8, 9]. Nonetheless, clinicians should remain aware that in case of non-response, alternative or adjunctive therapeutic interventions to attenuate catecholamine requirement in septic shock, such as corticosteroids [40] or angiotensin-II [41], can be considered to reduce catecholamine burden.

### Shock duration

Brask et al. noticed in a retrospective analysis that early AVP initiation (< 3 h NE) resulted in earlier shock resolution (38 vs. 61 h), with a significant decrease in NE 3 h after AVP initiation in the late group while this was minimal in the early group [20]. Nevertheless, the early AVP group received more fluids prior to vasopressor therapy which was not corrected for. Ragoonanan et al. observed in their propensity-score matched multicenter cohort no benefit in shock duration when starting AVP at NE < 0.25 µg/kg/min, while adding AVP within 4 h of NE therapy resulted in shorter shock duration [12]. Our observation that septic shock patients with longer NE infusion duration prior to AVP initiation were more likely to remain dependent of vasopressor therapy at any given time point during shock aligns with these results, but was less pronounced. Nevertheless, the links between higher baseline NE rates and prolonged vasopressor use, and low arterial pH with longer shock support early adjunctive AVP use in septic shock.

### Rebound Hypotension

According to a patient-level meta-analysis, stopping AVP before NE may increase the risk of hypotension compared to ceasing NE before AVP (61% vs. 43%)[42]. In our study, rebound hypotension, defined as an increase of NE requirement within 2 h of discontinuation of AVP before NE, occurred in only 9% of patients, the lowest reported to date [24, 42, 43]. Variations in definitions likely explain differences since most included studies evaluated hypotension at 24 h, whereas MAP declines more typically occur within the first 1–2 h after ceasing vasopressor therapy [43]. We found no association between rebound hypotension and NE infusion rate or AVP tapering, while AVP therapy > 24 h was associated with a lower likelihood of rebound. Sacha et al. observed a time-varying effect when AVP was discontinued before NE [43], suggesting that patients experiencing shock beyond 24 h may be more likely to have stabilized. The recovery of endogenous vasopressin levels, which decrease 6 h after septic shock onset [44] but increase at 24 h critical illness [45], may have contributed to this. However, the small number of events hampers the ability for definitive conclusions but may inform future prospective studies.

### Strengths and limitations

This study is the first prospective observational study outside the United States to evaluate hemodynamic characteristics following AVP initiation in septic shock patients. A key strength is our innovative approach to defining AVP responsiveness, which may better capture real-world hemodynamic responses. Additionally, assessing NE dynamics before AVP initiation, as proposed by Guerci et al. [11], is novel. Another strength is the reporting of NE doses as base equivalents, which is critical for standardizing comparisons in multicenter ICU studies [23]. However, one center initially reported NE as tartrate, and after conversion, three patients did not meet the 0.25 mcg/kg/min NE-base equivalent inclusion criteria, but were retained in analyses. Furthermore, additional testing within the same cohort may have induced type I error inflation and was not corrected for. Lastly, our study did not assess other characteristics that may affect hemodynamics during septic shock, including pre-ICU fluid resuscitation, sedatives, blood products, albumin levels, empirical antibiotic therapy or source control adequacy, which may have introduced confounding bias. These limitations emphasize the need to cautiously interpret our findings.

## Conclusions

In this multicenter study in septic shock patients treated with adjunct AVP, we observed a high hemodynamic response rate of 79% 2 h after AVP initiation, accompanied by a significant reduction in NE and NEE requirements. Obesity, NE dosage < 0.30 mcg/kg/min, and hyperlactatemia were negatively associated with AVP responsiveness, while NE infusion rate and duration, BMI and arterial pH at AVP initiation were associated to shock duration. Rebound hypotension was relatively rare, with a lower odds after prolonged administration of AVP. Our findings appear confirmative of previous observational studies and may help in selecting septic shock patients with a higher likelihood to show a hemodynamic response to AVP as a second-line vasopressor. Future prospective studies are needed to refine AVP use and determine optimal timing and dosing strategies in septic shock.

## Supplementary Information

Supplementary material 1

## Data Availability

The datasets used and/or analyzed during the current study are available from the corresponding author on reasonable request.

## Abbreviations

AVP: Arginine vasopressin
NE: Norepinephrine
ICU: Intensive care unit
MAP: Mean arterial pressure
RRT: Renal replacement therapy
SOFA: Sequential organ failure assessment
APACHE-IV: Acute physiological and chronic health evaluation
NEE: Norepinephrine equivalent
SD: Standard deviation
IQRL: Interquartile range
ROC: Receiver operating curve
BMI: Body mass index
ABW: Actual body weight
OR: Odd’s ratio
aOR: Adjusted odd’s ratio
AUC: Area under the curve
CI: Confidence
