# Supplementary material for: Hemodynamic effects of adjunct arginine vasopressin to norepinephrine in septic shock: insights from a prospective multicenter registry study
Source: Ann Intensive Care. 2025 Apr 29;15:59. doi: 10.1186/s13613-025-01472-w (PMC12040798; doi:10.1186/s13613-025-01472-w)
Supplement: Supplementary file 1 — Supplementary material 1 [file 13613_2025_1472_MOESM1_ESM.docx]

**Supplemental digital file content of: Hemodynamic effects of adjunct arginine vasopressin to norepinephrine in septic shock, insights from a prospective multicentre registry study**

[**Supplemental Figure 1:** Flowchart as a guideline to initiate, titrate, and taper AVP in patients with septic shock in the intensive care unit. 2](#_Toc193616678)

[**Supplemental Figure 2:** Expanded flowchart of the study population, including distribution among the participating centers in the study 3](#_Toc193616679)

[**Supplemental Figure 3:** Heatmap of missing baseline data 4](#_Toc193616680)

[**Supplemental Table 1**: Distribution of continuous baseline characteristics after multiple imputation 5](#_Toc193616681)

[**Supplemental Table 2:** Associations between baseline characteristics and AVP-responsiveness 6](#_Toc193616682)

[**Supplemental Table 3:**  Sensitivity analysis for a decrease in NE 2 hours after AVP initiation 7](#_Toc193616683)

[**Supplemental Figure 6:** NE infusion rates in responders, non-responders and the whole cohort 8](#_Toc193616684)

[**Supplemental Figure 5:** NEE infusion rates in responders, non-responders and the whole cohort. 9](#_Toc193616685)

[**Supplemental Figure 6:** Dynamics of NEE delta, lactate, MAP and Net Fluid Balance comparing patients with NE reduction to non-responders and those with a stabilization of NE at two hours after start of AVP. 10](#_Toc193616686)

[**Supplemental Figure 7:** NE infusion rates (% of baseline) in responders, non-responders and the whole cohort. 11](#_Toc193616687)

[**Supplemental Figure 8:** NEE (% of baseline) in responders, non-responders and the whole cohort. 12](#_Toc193616688)

[**Supplemental Figure 9:** Arterial pH levels at baseline and after start of AVP. 13](#_Toc193616689)

[**Supplemental Table 4:** Probability of longer shock duration in 140 shock survivors 14](#_Toc193616690)

[**Supplemental Table 5:** Associations between clinical characteristics and rebound hypotension in 129 shock survivors 15](#_Toc193616691)

#
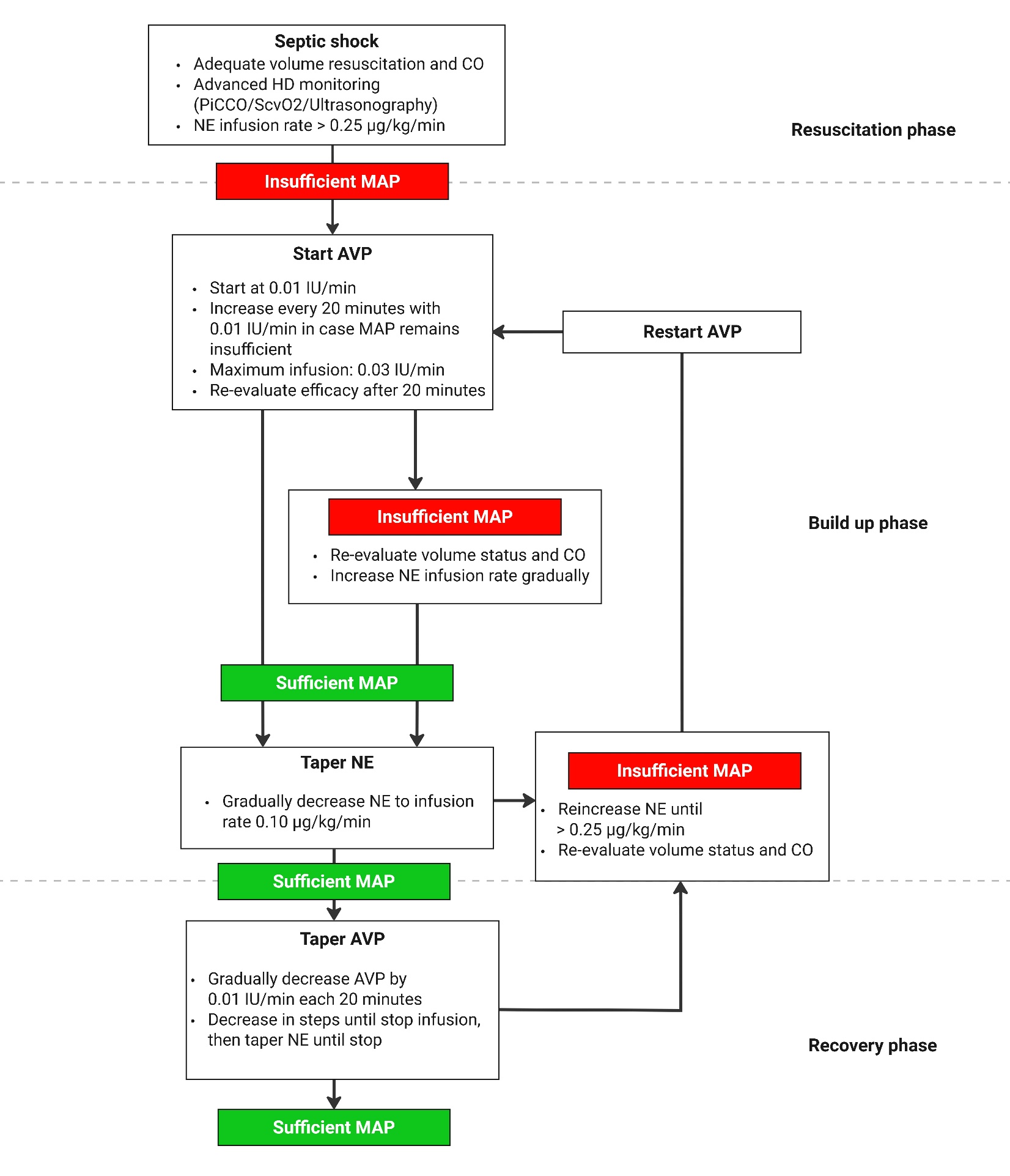
Supplemental Figure 1: Flowchart as a guideline to initiate, titrate, and taper AVP in patients with septic shock in the intensive care unit.

This flowchart was distributed to all participating centers as a guideline for treating septic shock patients with AVP as an adjunct vasopressor.
|Abbreviations: PiCCO = Pulse Contour Cardiac Output; ScVO_2_ = Central Venous Oxygen Saturation; CO = Cardiac Output; AVP = Arginine vasopressin; MAP = Mean Arterial Pressure

# Supplemental Figure 2: Expanded flowchart of the study population, including distribution among the
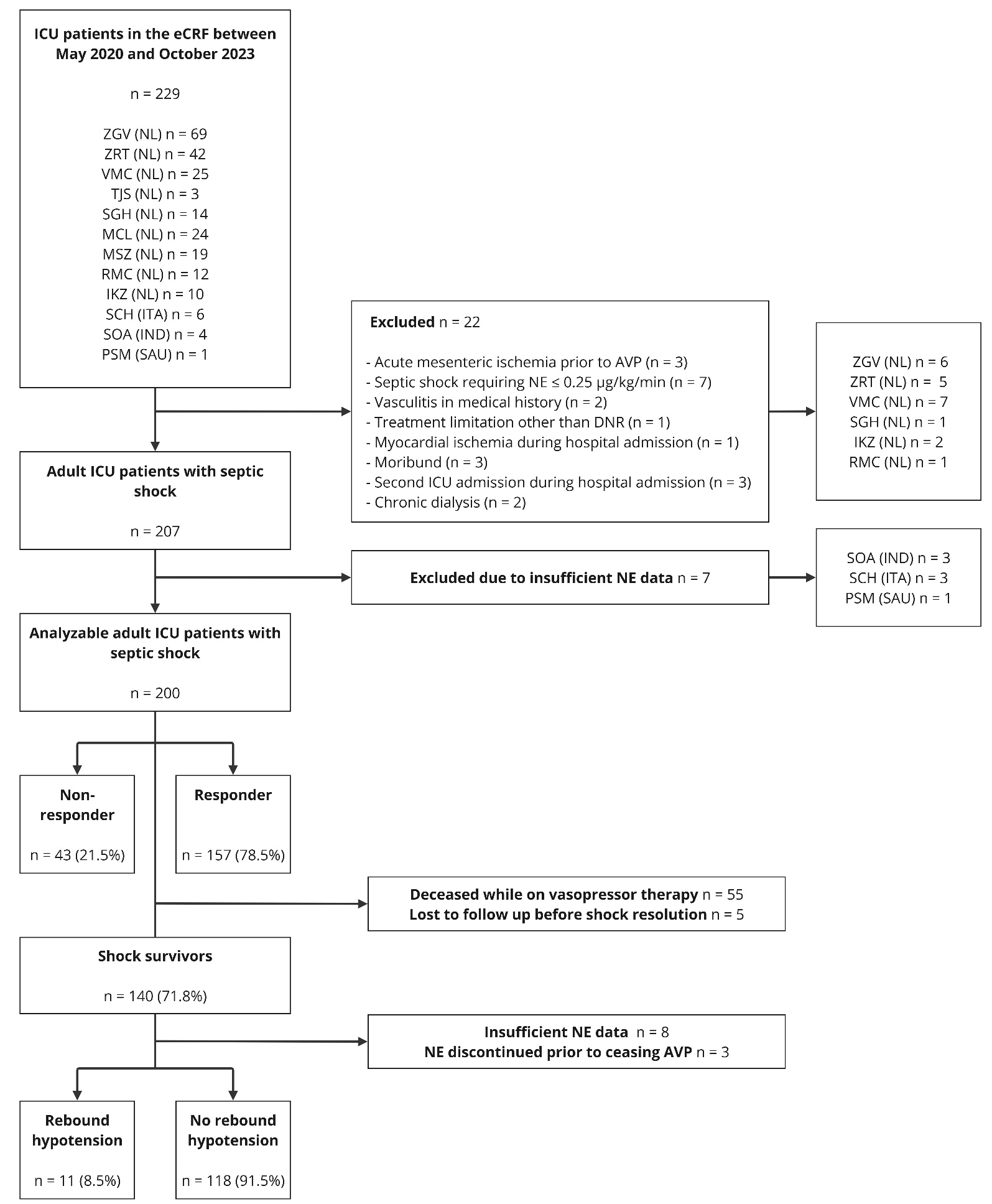
participating centers in the study

Abbreviations: SOA = Siksha ‘O’ Anusandhan Hospital; SCH = San Carlo Hospital; MCL = Medical Centre Leeuwarden; MSZ = Maasstad Hospital; RMC = Radboud University Medical Center; SGH = Spaarne Gasthuis Hospital; TJS = Tjongerschans Hospital; VMC = Viecuri Medical Centre; ZGV = Gelderse Vallei Hospital; ZRT = Rivierenland Hospital; PSM = Prince Sultan Medical Centre; NL = the Netherlands; ITA = Italy, IND = India; SAU = Saudi Arabia; DNR = Do-not-resuscitate; RRT = Renal Replacement Therapy; ICU = Intensive Care Unit; NE = Norepinephrine; AVP = Arginine Vasopressin

#
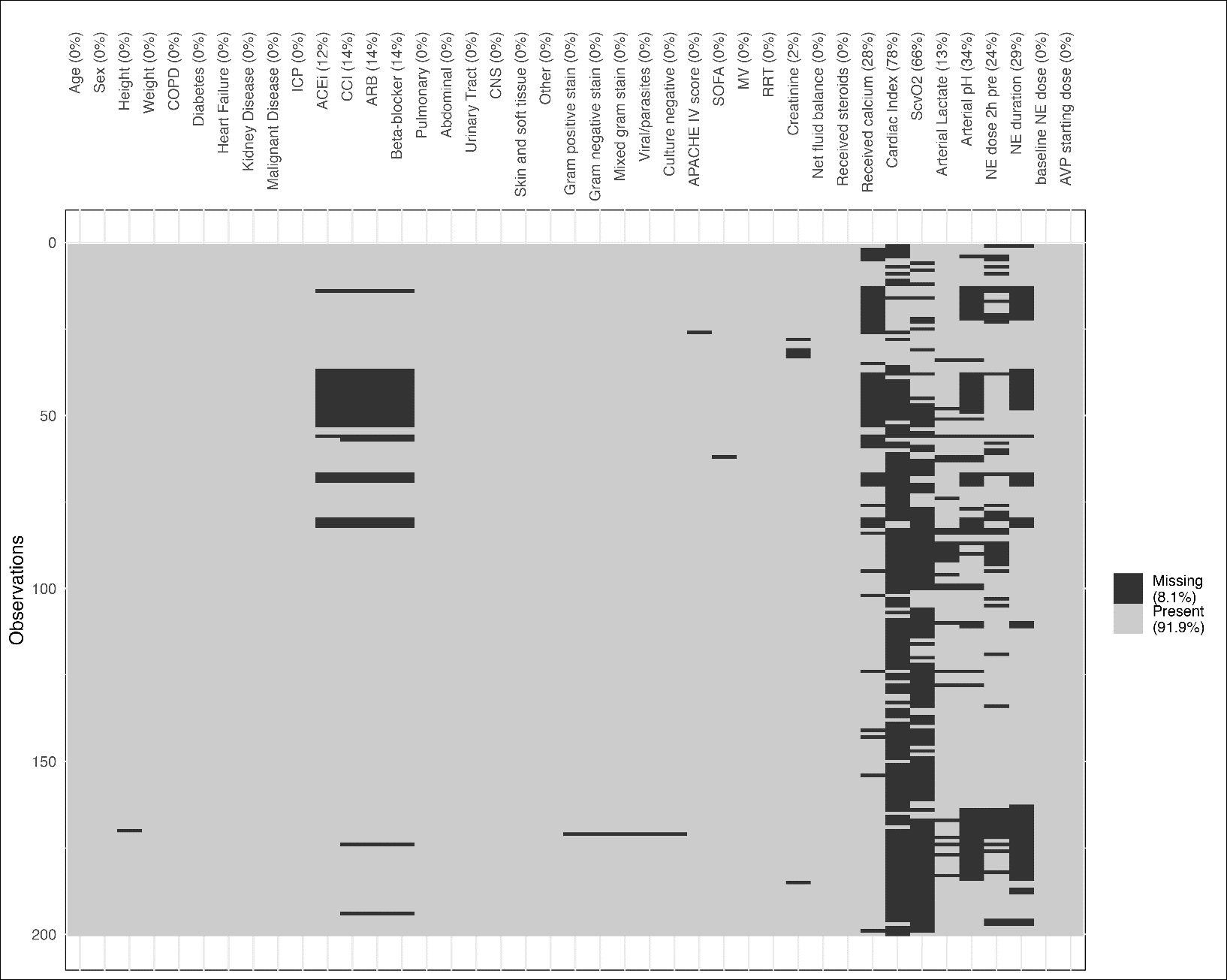
Supplemental Figure 3: Heatmap of missing baseline data

Hawkin’s non-parametric test for continuous missing data was non-significant (*p* = 0.260), indicative of MCAR among continuous missing data with missing rates less than 40%. Abbreviations: MCAR = Missing Completely At Random; COPD = Chronic Obstructive Pulmonary Disease; ACEi = Angiotensin Converting Enzyme inhibitor; CCI = Calcium Channel inhibitor; ARB = Angiotensin Receptor Blocker; CNS = Central Nerve System; APACHE = Acute Physiological and Chronic Health Evaluation; SOFA = Sequential Organ Failure; MV = Mechanical Ventilation; RRT = Renal Replacement therapy; ScvO2 = Central Venous Oxygen Saturation ; NE = Norepinephrine; AVP = Arginine Vasopressin

| **Characteristics** | **Total**  ***n* = 200** | **Non-responder ^5^**  ***n* = 43** | **Responder ^5^**  ***n* = 157** | ***p*-value** |
| --- | --- | --- | --- | --- |
| BMI on admission, kg/m^2^ | 27.0 [24.0 – 32.0] | 30.4 [26.8 – 34.8] | 26.2 [23.7 – 31.0] | <0.001 |
| IBW, kg | 58.5 [47.9 – 66.9] | 59.2 [50.8 – 71.0] | 58.2 [7.0 – 66.6] | 0.128 |
| **Shock related characteristics** |  |  |  |  |
| APACHE IV score | 92 [75 – 112] | 93 [77 – 117] | 92 [75 – 111] | 0.575 |
| SOFA score | 10 [8 – 12] | 10 [8 – 12] | 10 [8 – 12] | 0.930 |
| Creatinine, µmol/L | 102 [74 – 166] | 98 [72 – 177] | 105 [76 – 166] | 0.919 |
| Arterial lactate, mmol/L | 3.2 [2.1 – 5.2] | 3.7 [2.4 – 6.6] | 2.9 [2.1 – 4.5] | 0.096 |
| Arterial pH | 7.29 [7.22 – 7.34] | 7.28 [7.23 – 7.32] | 7.29 [7.22 – 7.35] | 0.735 |
| NE duration, hours | 7 [4 – 16] | 6 [3 – 9] | 8 [4 – 17] | 0.183 |
| NEE delta 2 h prior to baseline, % | 18 [5 – 45] | 20 [6 – 51] | 17 [5 – 44] | 0.418 |

# Supplemental Table 1: Distribution of continuous baseline characteristics after multiple imputation

| **Baseline variables** | **Univariate Logistic Regression** | | **Multivariable Logistic Regression ^1^** | |
| --- | --- | --- | --- | --- |
|  | OR (95% CI) | *p*-value | OR (95% CI) | *p*-value |
| **Age (years)** | 1.012 (0.988 – 1.037) | 0.331 |  |  |
| **Sex (male)** | 1.010 (0.512 – 1.992) | 0.978 |  |  |
| **Weight (kg)** | 0.981 (0.967 – 0.995) | 0.009 |  |  |
| **IBW (kg) ^2^** | 0.981 (0.957 – 1.006) | 0.144 |  |  |
| **BMI (kg/m^2^)** | 0.941 (0.899 – 0.984) | 0.008 |  |  |
| **Obesity ^3^** | 0.310 (0.154 – 0.623) | <0.001 | 0.303 (0.142 – 0.646) | 0.002 |
| **Diabetes** | 0.685 (0.302 – 1.556) | 0.367 |  |  |
| **Immunocompromised ^4^** | 0.955 (0.297 – 3.064) | 0.938 |  |  |
| **Anti-hypertensive medication ^5^** | 1.726 (0.776 – 3.840) | 0.181 |  |  |
| **Abdominal sepsis** | 0.637 (0.323 – 1.256) | 0.193 |  |  |
| **Pulmonary sepsis** | 1.634 (0.748 – 3.571) | 0.218 |  |  |
| **SOFA-score** | 1.007 (0.904 – 1.122) | 0.901 |  |  |
| **APACHE IV score** | 0.999 (0.987 – 1.010) | 0.799 |  |  |
| **Mechanical Ventilation** | 1.334 (0.572 – 3.110) | 0.505 |  |  |
| **RRT** | 4.766 (0.614 – 37.001) | 0.135 | 7.987 (0.933 – 68.365) | 0.058 |
| **Creatinine (µmol/L)** | 1.000 (0.996 – 1.003) | 0.832 |  |  |
| **Net Fluid balance (L)** | 0.979 (0.905 – 1.059) | 0.598 |  |  |
| **Net Fluid balance (per 100 ml/kg IBW)** | 0.999 (0.952 – 1.048) | 0.961 |  |  |
| **Received steroids** | 1.025 (0.514 – 2.043) | 0.944 |  |  |
| **Received calcium intravenous** | 1.546 (0.419 – 5.705) | 0.513 |  |  |
| **ScVO_2_ (%)** | 0.971 (0.909 – 1.039) | 0.394 |  |  |
| **Arterial lactate (per mmol/L)** | 0.910 (0.801 – 1.033) | 0.145 | 0.861 (0.746 – 0.994) | 0.041 |
| **Arterial pH (per 0.1)** | 1.040 (0.719 – 1.503) | 0.837 |  |  |
| **NE duration, hours** | 1.001 (0.989 – 1.014) | 0.841 |  |  |
| **NE dosage, per 0.1 mcg/kg/min** | 1.172 (0.989 – 1.389) | 0.066 |  |  |
| **NE dosage, mcg/min** | 1.002 (0.985 – 1.02-) | 0.795 |  |  |
| **NE dosage ≥ 0.30 mcg/kg/min** | 3.012 (1.444 – 6.284) | 0.003 | 2.331 (1.058 – 5.137) | 0.036 |
| **NEE delta 2 h prior to baseline** | 0.996 (0.987 – 1.006) | 0.426 |  |  |
| **AVP dosage (IU/min)** | 1.003 (0.566 – 1.775) | 0.993 |  |  |

# Supplemental Table 2: Associations between baseline characteristics and AVP-responsiveness

^1^ Variables in multivariable model selected using Least Absolute Shrinkage and Selection Operator (LASSO) regression at λ_min_. 200 patients were included in the multivariable model; ^2^ Obtained using the Gallagher formula (23); ^3^ BMI ≥ 30 kg/m^2^; ^4^ Long-term use of immunosuppressive therapy or use of corticosteroids (e.g. >5 days 1mg/kg prednisone or 20 days ≥0.1mg/kg) or active chemo-or radiation therapy last year, or treatment for a lymphoma any time before ICU admission or documented humoral or cellular deficiencies; ^5^ includes the use of calcium channel inhibitor, angiotensin-converting-enzyme inhibitor and/or angiotensin receptor blocker within 48 hours from ICU admission

Abbreviations: IBW = Ideal Body Weight; BMI = Body Mass Index; SOFA = Sequential Organ Failure Assessment; APACHE = Acute Physiology and Chronic Health Evaluation; RRT = Renal Replacement Therapy; NE = Norepinephrine; NEE = Norepinephrine Equivalent; AVP = Arginine-Vasopressin

Supplemental Table 3: Sensitivity analysis for a decrease in NE 2 hours after AVP initiation

^1^ Variables in multivariable model selected using Least Absolute Shrinkage and Selection Operator (LASSO) regression at λ_min_. 200 patients were included in the multivariable model; ^2^ Obtained using the Gallagher formula (23); ^3^ BMI ≥ 30 kg/m^2^; ^4^ Long-term use of immunosuppressive therapy or use of corticosteroids (e.g. >5 days 1mg/kg prednisone or 20 days ≥0.1mg/kg) or active chemo-or radiation therapy last year, or treatment for a lymphoma any time before ICU admission or documented humoral or cellular deficiencies; ^5^ includes the use of calcium channel inhibitor, angiotensin-converting-enzyme inhibitor and/or angiotensin receptor blocker within 48 hours from ICU admission

Abbreviations: IBW = Ideal Body Weight; BMI = Body Mass Index; SOFA = Sequential Organ Failure Assessment; APACHE = Acute Physiology and Chronic Health Evaluation; RRT = Renal Replacement Therapy; NE = Norepinephrine; NEE = Norepinephrine Equivalent; AVP = Arginine-Vasopressin

| **Baseline variables** | **Univariate Logistic Regression** | | **Multivariable Logistic Regression ^1^** | |
| --- | --- | --- | --- | --- |
|  | OR (95% CI) | *p*-value | OR (95% CI) | *p*-value |
| **Age (years)** | 1.038 (1.014 – 1.062) | 0.002 | 1.034 (1.008 – 1.061) | 0.011 |
| **Sex (male)** | 1.003 (0.573 – 1.758) | 0.991 |  |  |
| **Weight (kg)** | 0.981 (0.968 – 0.995) | 0.008 |  |  |
| **IBW (kg) ^2^** | 0.982 (0.960 – 1.003) | 0.096 |  |  |
| **BMI (kg/m^2^)** | 0.944 (0.903 – 0.987) | 0.011 |  |  |
| **Obesity ^3^** | 0.410 (0.219 – 0.767) | 0.005 | 0.573 (0.289 – 1.137) | 0.111 |
| **Diabetes** | 1.744 (0.848 – 3.584) | 0.130 |  |  |
| **Immunocompromised ^4^** | 0.429 (0.147 – 1.254) | 0.122 |  |  |
| **Anti-hypertensive medication ^5^** | 1.917 (1.017 – 3.612) | 0.044 |  |  |
| **Abdominal sepsis** | 1.198 (0.686 – 2.093) | 0.526 |  |  |
| **Pulmonary sepsis** | 0.892 (0.488 – 1.631) | 0.710 |  |  |
| **SOFA-score** | 0.924 (0.843 – 1.013) | 0.093 |  |  |
| **APACHE IV score** | 0.996 (0.986 – 1.005) | 0.361 |  |  |
| **Mechanical Ventilation** | 1.517 (0.716 – 3.214) | 0.276 |  |  |
| **RRT** | 0.629 (0.223 – 1.773) | 0.380 |  |  |
| **Creatinine (µmol/L)** | 0.993 (0.989 – 0.997) | <0.001 |  |  |
| **Net Fluid balance (L)** | 1.010 (0.944 – 1.081) | 0.766 |  |  |
| **Net Fluid balance (per 100 ml/kg IBW)** | 1.018 (0.979 – 1.059) | 0.381 |  |  |
| **Received steroids** | 0.633 (0.356 – 1.124) | 0.118 |  |  |
| **Received calcium intravenous** | 1.228 (0.457 – 3.298) | 0.684 |  |  |
| **ScVO_2_ (%)** | 1.044 (0.990 – 1.100) | 0.110 |  |  |
| **Arterial lactate (per mmol/L)** | 0.925 (0.823 – 1.039) | 0.190 | 0.888 (0.783 – 1.008) | 0.066 |
| **Arterial pH (per 0.1)** | 0.988 (0.728 – 1.341) | 0.938 |  |  |
| **NE duration, hours** | 0.997 (0.986 – 1.007) | 0.516 |  |  |
| **NE dosage, per 0.1 mcg/kg/min** | 1.159 (1.028 – 1.306) | 0.016 |  |  |
| **NE dosage, mcg/min** | 1.007 (0.993 – 1.021) | 0.343 |  |  |
| **NE dosage ≥ 0.30 mcg/kg/min** | 6.423 (2.702 – 15.267) | <0.001 | 5.126 (2.096 – 12.533) | <0.001 |
| **NEE delta 2 h prior to baseline** | 1.010 (0.998 – 1.023) | 0.095 |  |  |
| **AVP dosage (IU/min)** | 0.674 (0.415 – 1.093) | 0.110 |  |  |


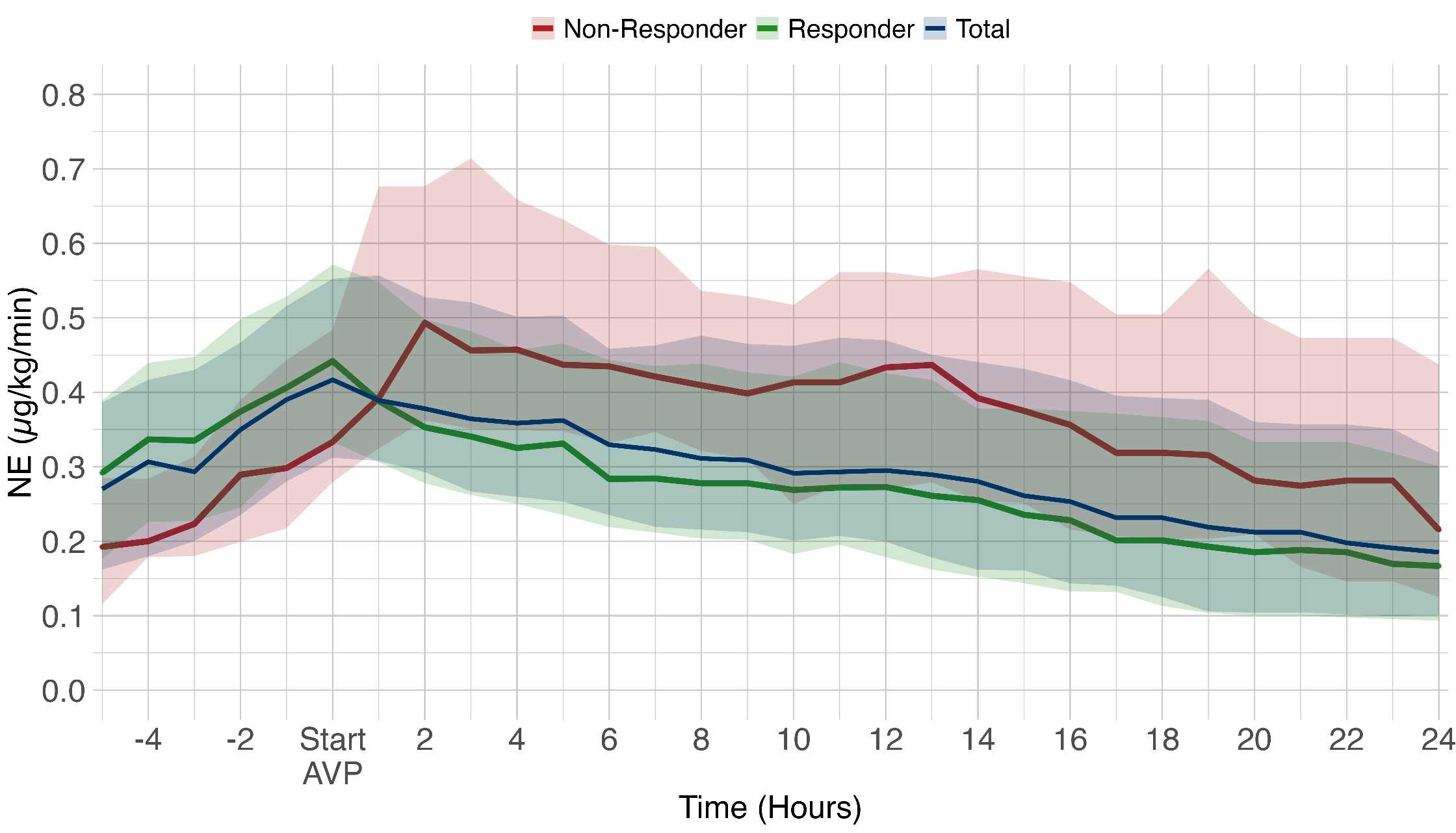
Supplemental Figure 6: NE infusion rates in responders, non-responders and the whole cohort.

In the LMM evaluating NE trajectory five hours prior to AVP initiation, the interaction between response and time was significant (*p*=0.024), indicating a larger increase in NE requirement in non-responders. However, this was no longer significant after applying robust standard errors for substantial heteroscedasticity (*p*=0.073)*.* In the whole cohort, the NE infusion rate was significantly lower two and five hours after the start of AVP (0.42 µg/kg/min [0.31 – 0.55] vs. 0.38 µg/kg/min [0.29 – 0.53], *p* < 0.001, and 0.36 µg/kg/min [0.25 – 0.50], *p* < 0.001, respectively). Medians alongside interquartile ranges are presented. Abbreviations: AVP = Arginine Vasopressin; NE = Norepinephrine

#
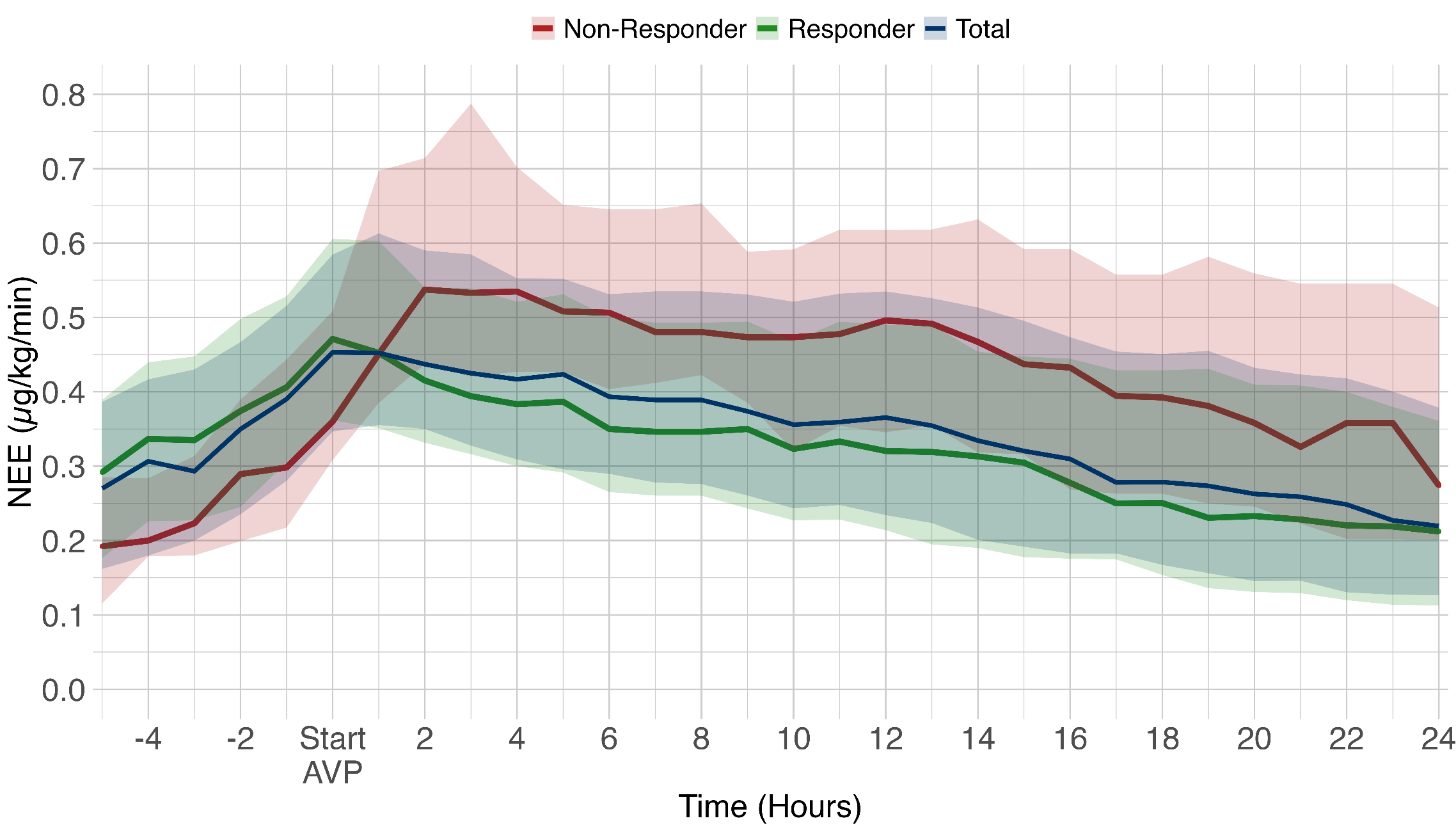
Supplemental Figure 5: NEE infusion rates in responders, non-responders and the whole cohort.

Within the whole cohort, NEE was not significantly lower compared to baseline at two hours after AVP initiation (0.46 µg/kg/min [0.34 – 0.60] vs. 0.44 µg/kg/min [0.35 – 0.59], *p* = 0.718), while NEE at five hours it was significantly lower (0.42 µg/kg/min [0.29 – 0.55], *p* < 0.001). Medians alongside interquartile ranges are presented. Abbreviations: AVP = Arginine Vasopressin; NEE = Norepinephrine Equivalent

#
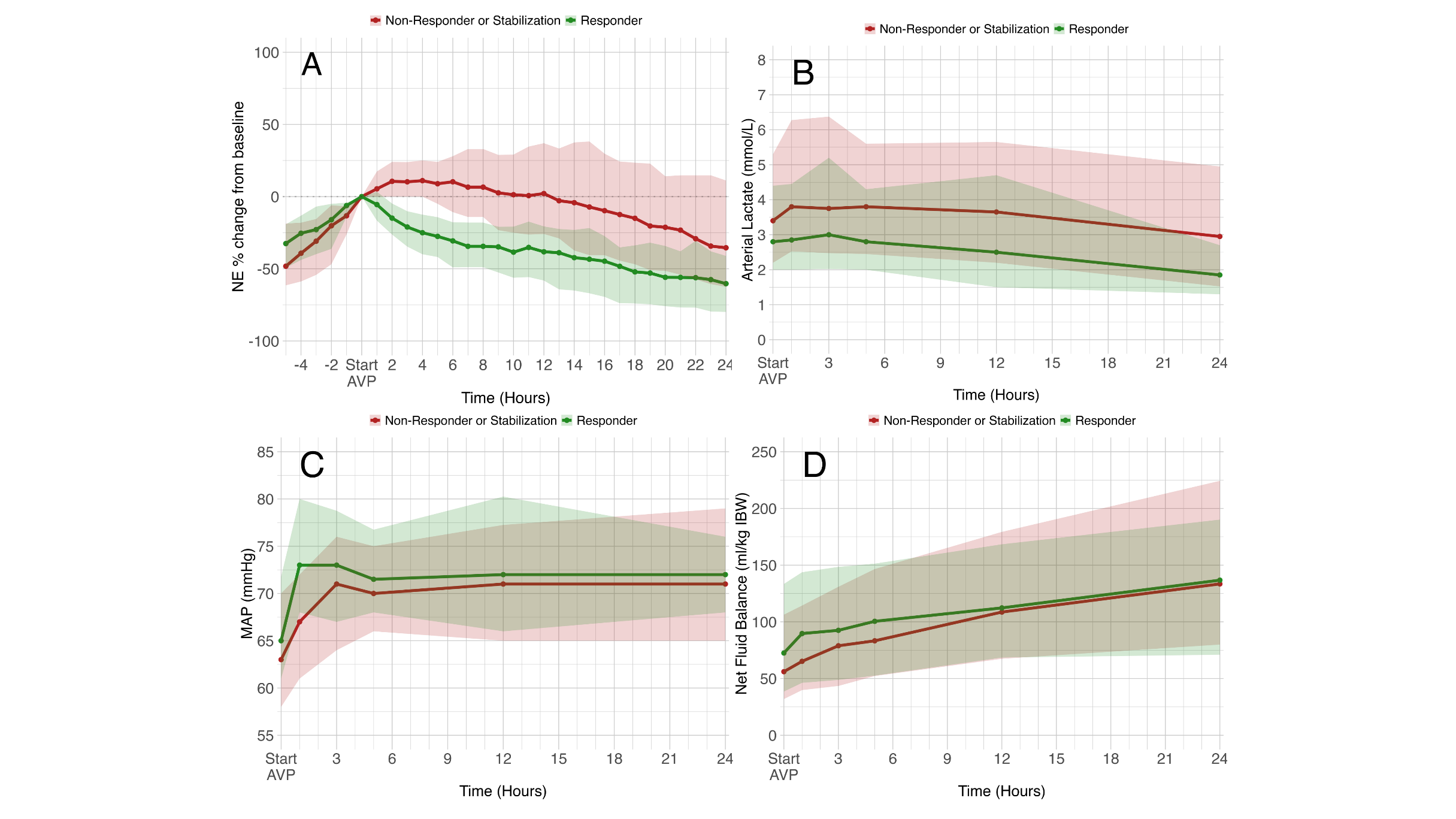
Supplemental Figure 6: Dynamics of NEE delta, lactate, MAP and Net Fluid Balance comparing patients with NE reduction to non-responders and those with stabilization of NE at two hours after the start of AVP.

**A.** **NEE dynamics before and after the start of AVP.** An LMM showed no significant differences in NEE at five hours before the start of AVP between those with NE reduction two hours after start of AVP compared to others (p=0.588), nor was there a significant difference in trajectory five hours prior to AVP start (*p*=0.702)

**B.** **Arterial lactate levels at baseline and after the start of AVP.** An LMM showed no significant baseline differences in arterial lactate in patients with NE reduction compared to others (*p*=0.094) but a higher lactate increase than others (*p*=0.030). After applying robust standard errors due to heteroscedasticity, the interaction between NE reduction and time was no longer significant (*p*=0.069).

**C. Mean Arterial Pressure (MAP) at baseline and after the start of AVP.** An LMM showed a significantly higher baseline in patients with NE reduction compared to non-responders and those with a stabilization of NE (average 4.5 mmHg higher, *p*<0.001) with a higher increase in MAP over time in patients with NE reduction (average increase 0.21 mmHg, *p*=0.016).

**D. Net fluid balance at baseline and after the start of AVP** An LMM showed no significant baseline differences between patients with NE reduction at two hours compared to others (*p*=0.345), but this increased less over time (average 0.92 ml/kg IBW per hour less, *p*=0.016) which remained significant after applying robust standard errors (*p*=0.015).

In all figures, medians alongside interquartile ranges are presented. Abbreviations: NEE = Norepinephrine Equivalent; AVP = Arginine vasopressin; MAP = Mean Arterial Pressure; IBW = Ideal Body Weight; LMM = Linear Mixed Model

#
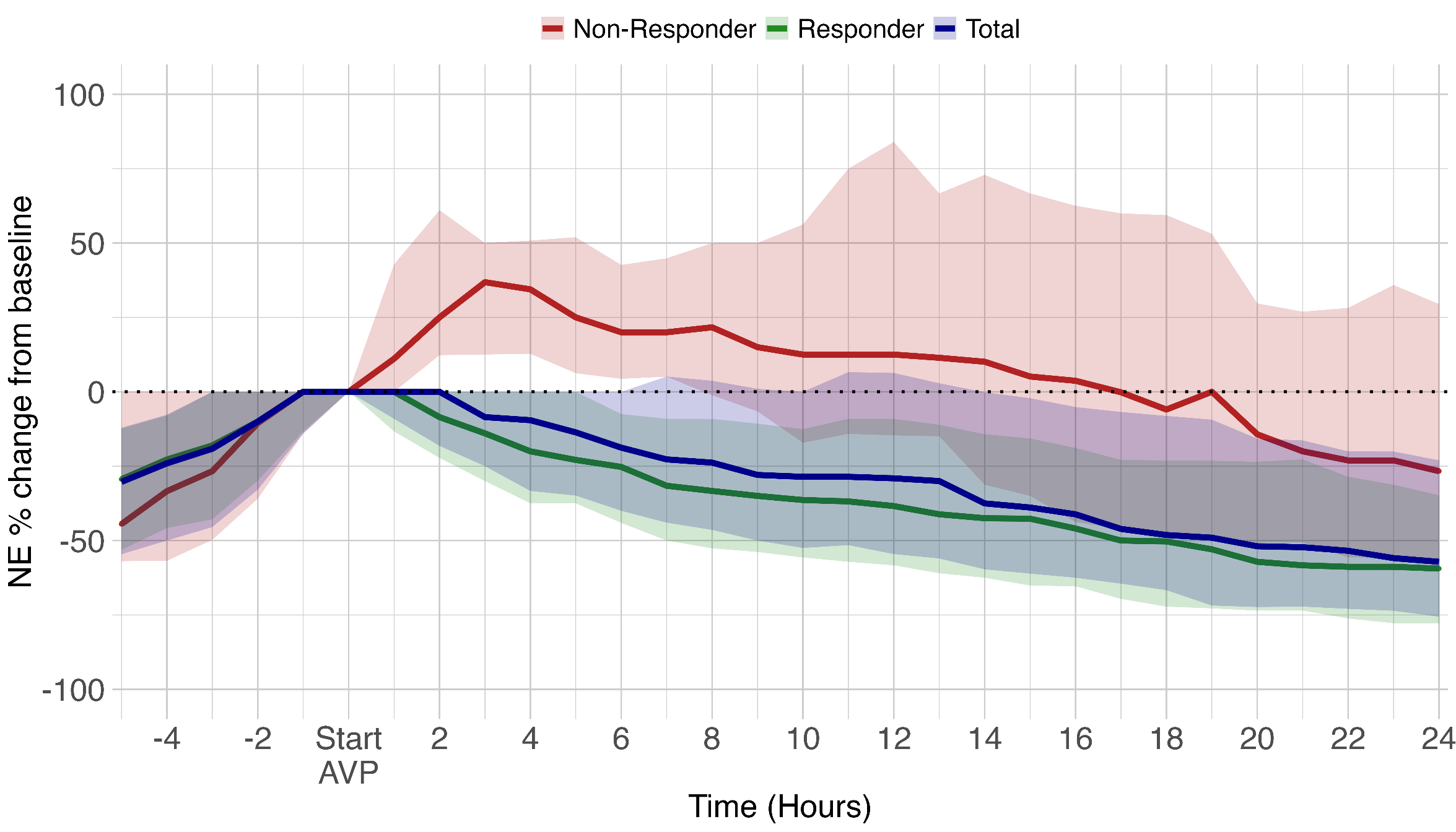
Supplemental Figure 7: NE infusion rates (% of baseline) in responders, non-responders and the whole cohort.

Within the whole cohort, the NE percentage of baseline at two hours and five hours was significantly lower compared to baseline (0% [-18 – 0], *p* = 0.008, and -14% [-35 – 0], *p* < 0.001, respectively). Medians alongside interquartile ranges are presented. Abbreviations: AVP = Arginine Vasopressin; NE = Norepinephrine

#
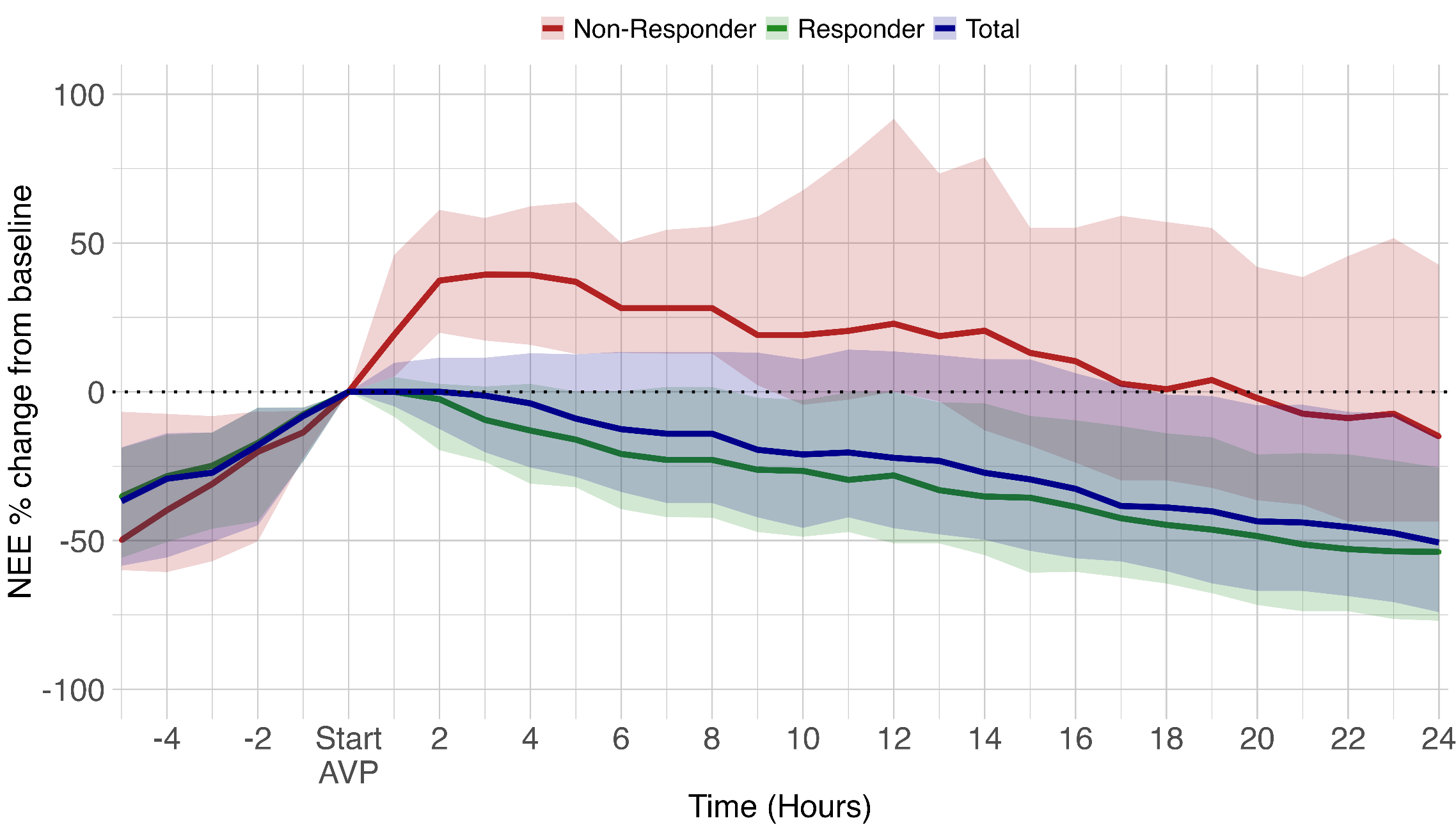
Supplemental Figure 8: NEE (% of baseline) in responders, non-responders and the whole cohort.

Within the whole cohort, the NEE percentage of baseline was not lower at two hours after AVP initiation (0% [-13 – 11], *p* = 0.933), while at five hours, it was significantly lower than baseline (-9% [-31 – 11],  *p* = 0.001). Medians alongside interquartile ranges are presented. Abbreviations: AVP = Arginine Vasopressin; NEE = Norepinephrine Equivalent

#
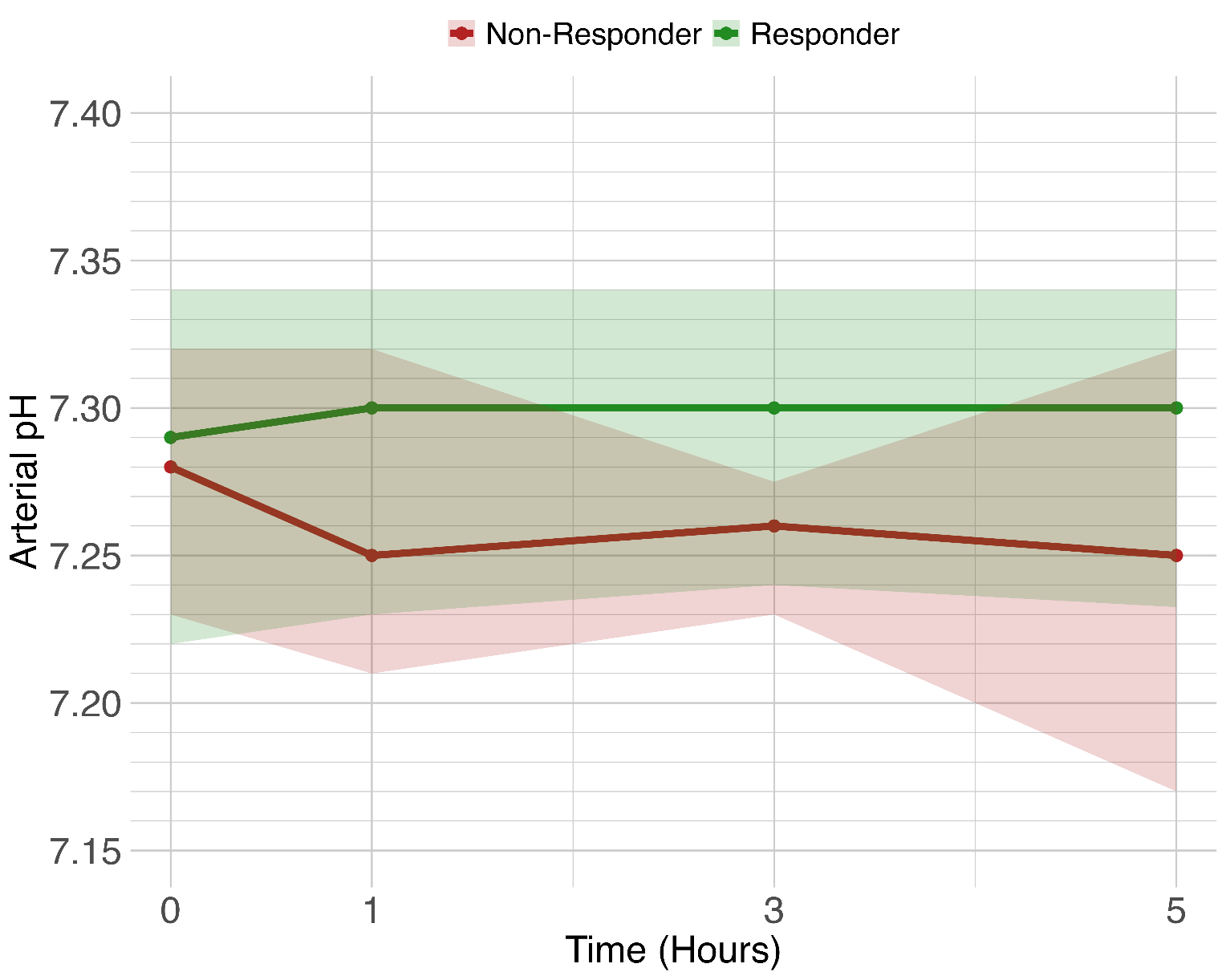
**Supplemental Figure 9**: Arterial pH levels at baseline and after the start of AVP.

An linear mixed-effects model was conducted to test the change in arterial pH over time. While arterial pH did not significantly decrease over time overall (*p*=0.123), nor were there significant differences at baseline in arterial pH between responders and non-responders (*p*=0.553), arterial pH decreased 0.01 per hour faster in non-responders than responders (*p*=0.005) which remained significant after applying robust standard errors (*p*=0.05) due to heteroscedasticity; Abbreviation: AVP = Arginine Vasopressin

| **Baseline variables** | **Univariate Cox Regression** | | **Multivariable Cox Regression** **^1^** | |
| --- | --- | --- | --- | --- |
|  | OR (95% CI) | *p*-value | OR (95% CI) | *p*-value |
| **Age (years)** | 1.004 (0.982, 1.101) | 0.636 |  |  |
| **Sex (male)** | 1.120 (0.801, 1.565) | 0.509 |  |  |
| **Weight (kg)** | 1.009 (1.001, 1.017) | 0.024 |  |  |
| **IBW (kg) ^2^** | 1.009 (0.978, 1.004) | 0.170 |  |  |
| **BMI (kg/m^2^)** | 1.032 (1.007, 1.057) | 0.010 | 1.060 (1.017, 1.105) | 0.006 |
| **Obesity ^3^** | 1.426 (0.999, 2.037) | 0.051 |  |  |
| **Diabetes** | 1.277 (0.820, 1.988) | 0.278 |  |  |
| **Immunocompromised ^4^** | 1.596 (0.878, 2.899) | 0.126 |  |  |
| **Congestive Heart failure** | 0.738 (0.374, 1.458) | 0.381 |  |  |
| **Anti-hypertensive medication ^5^** | 0.866 (0.584, 1.282) | 0.472 |  |  |
| **Abdominal sepsis** | 0.765 (0.547, 1.068) | 0.116 |  |  |
| **Pulmonary sepsis** | 1.117 (0.772, 1.616) | 0.557 |  |  |
| **Urinary tract sepsis** | 0.754 (0.382, 1.491) | 0.417 |  |  |
| **SOFA-score** | 1.066 (0.999, 1.138) | 0.054 |  |  |
| **APACHE IV score** | 1.003 (0.997, 1.008) | 0.343 |  |  |
| **Mechanical Ventilation** | 1.001 (0.647, 1.548) | 0.997 |  |  |
| **RRT** | 1.776 (0.930, 3.401) | 0.082 |  |  |
| **Creatinine (µmol/L)** | 1.002 (0.999, 1.004) | 0.146 |  |  |
| **Net fluid balance (L)** | 1.061 (1.015, 1.107) | 0.008 | 1.025 (0.972, 1.080) | 0.369 |
| **Net fluid balance (per 100 ml/kg IBW^1^)** | **1.003 (1.001, 1.006)** | 0.016 |  |  |
| **Received steroids** | 1.852 (0.981, 3.485) | 0.446 |  |  |
| **Received calcium intravenous** | 1.852 (0.981, 3.485) | 0.057 |  |  |
| **ScVO_2_ (%)** | 1.004 (0.970, 1.041) | 0.808 |  |  |
| **Arterial lactate (mmol/L)** | 1.028 (0.950, 1.112) | 0.500 |  |  |
| **Arterial pH (per 0.1)** | 0.725 (0.588, 0.893) | 0.002 | 0.799 (0.640, 0.999) | 0.049 |
| **NE duration (per hour)** | 1.017 (1.006, 1.029) | 0.003 | 1.013 (1.000, 1.026) | 0.050 |
| **NE duration ≥ 7 hours** | 1.524 (1.082, 2.146) | 0.016 |  |  |
| **NE dosage (per 0.1 mcg/kg/min)** | 1.059 (0.970, 1.156) | 0.202 | 1.122 (1.009, 1.248) | 0.033 |
| **NE dosage (mcg/min)** | 1.017 (1.004, 1.029) | 0.008 |  |  |
| **NE dosage ≥ 0.30 mcg/kg/min** | 1.168 (0.800, 1.707) | 0.419 |  |  |
| **NEE delta 2 h prior to baseline** | 0.999 (0.994 – 1.006) | 0.697 |  |  |
| **AVP dosage (IU/min)** | 1.147 (0.830, 1.582) | 0.406 |  |  |
| **AVP responsiveness ^6^** | 0.862 (0.565, 1.314) | 0.488 |  |  |

# Supplemental Table 4: Probability of longer shock duration in 140 shock survivors

^1^ Variables in multivariable model selected using Least Absolute Shrinkage and Selection Operator (LASSO) regression at λ_min._ 140 shock survivors were included in the multivariable model; ^2^ Obtained using the Gallagher formula (23); ^3^ BMI ≥ 30 kg/m^2^; ^4^ Long-term use of immunosuppressive therapy or use of corticosteroids (e.g. >5 days 1mg/kg prednisone or 20 days ≥0.1mg/kg) or active chemo-or radiation therapy last year, or treatment for a lymphoma any time before ICU admission or documented humoral or cellular deficiencies; ^5^ includes use of calcium channel inhibitor, angiotensin-converting-enzyme inhibitor and/or angiotensin receptor blocker within 48 hours from ICU admission; ^6^ Decrease or stabilization of NE-requirement 2 hours after initiation of AVP. Abbreviations: IBW = Ideal Body Weight; BMI = Body Mass Index; SOFA = Sequential Organ Failure Assessment; APACHE = Acute Physiology and Chronic Health Evaluation; RRT = Renal Replacement Therapy; NE = Norepinephrine; NEE = Norepinephrine Equivalent; AVP = Arginine-Vasopressin

| **Characteristics** | **Univariate Logistic Regression** | |
| --- | --- | --- |
|  | OR (95% CI) | *p*-value |
| Age (years) | 1.067 (1.002, 1.137) | 0.044 |
| Sex (male) | 0.482 (0.134, 1.735) | 0.264 |
| Weight (kg) | 0.996 (0.967, 1.026) | 0.778 |
| IBW (kg) ^1^ | 0.971 (0.921, 1.024) | 0.275 |
| BMI (kg/m^2^) | 1.005 (0.924, 1.094) | 0.900 |
| Obesity ^2^ | 1.158 (0.320, 4.192) | 0.824 |
| Diabetes | 3.395 (0.896, 12.857) | 0.072 |
| Immunocompromised ^3^ | - | - |
| Heart failure | 1.867 (0.204, 17.078) | 0.581 |
| Anti-hypertensive medication ^4^ | 1.544 (0.439, 5.433) | 0.498 |
| Abdominal sepsis | 1.937 (0.538, 6.972) | 0.311 |
| Pulmonary sepsis | 0.549 (0.113, 2.674) | 0.458 |
| SOFA-score at baseline | 0.934 (0.727, 1.201) | 0.596 |
| APACHE IV score | 1.013 (0.990, 1.037) | 0.260 |
| **Shock-related characteristics** |  |  |
| AVP-responsiveness ^5^ | 0.544 (0.133, 2.232) | 0.398 |
| AVP tapered ^6^ | 0.446 (0.047, 4.203) | 0.481 |
| NE dosage (per 0.1 mcg/kg/min) ^7^ | 0.916 (0.589, 1.420) | 0.169 |
| NE dosage (mcg/min) ^7^ | 0.970 (0.897, 1.049) | 0.443 |
| NE dosage ≥ 0.10 mcg/kg/min ^7^ | 0.482 (0.134, 1.735) | 0.264 |
| NE dosage ≥ 0.20 mcg/kg/min ^7^ | 0.413 (0.050, 3.392) | 0.410 |
| AVP duration (hours) | 0.968 (0.934, 1.003) | 0.071 |
| AVP duration > 24 h | 0.215 (0.054, 0.854) | 0.029 |
| AVP cumulative dosage (IU) | 0.994 (0.979, 1.009) | 0.440 |

# Supplemental Table 5: Associations between clinical characteristics and rebound hypotension in 129 shock survivors

^1^ Obtained using Gallagher formula (23); ^2^ BMI ≥ 30 kg/m^2^; ^3^Long term use of immunosuppressive therapy or use of corticosteroids (e.g. >5 days 1mg/kg prednisone or 20 days ≥0.1mg/kg) or active chemo-or radiation therapy last year, or treatment for a lymphoma any time before ICU admission or documented humoral or cellular deficiencies; ^4^ includes use of calcium channel inhibitor, angiotensin-converting-enzyme inhibitor and/or angiotensin receptor blocker within 48 hours from ICU admission; ^5^ Decrease or stabilization of NE-requirement 2 hours after initiation of AVP; ^6^ stepwise decrease in AVP dosage one hour before ceasing AVP; ^7^ on the moment AVP is stopped.
Abbreviations: IBW = Ideal Body Weight; BMI = Body Mass Index; SOFA = Sequential Organ Failure Assessment; APACHE = Acute Physiology and Chronic Health Evaluation; RRT = Renal Replacement Therapy; NE = Norepinephrine; NEE = Norepinephrine Equivalent; AVP = Arginine-Vasopressin
